# Modifications to the net knee moments lead to the greatest improvements in accelerative sprinting performance: a predictive simulation study

**DOI:** 10.1038/s41598-022-20023-y

**Published:** 2022-09-23

**Authors:** Nicos Haralabidis, Steffi L. Colyer, Gil Serrancolí, Aki I. T. Salo, Dario Cazzola

**Affiliations:** 1 Department for Health, University of Bath, Claverton Down, Bath, BA2 7AY UK; 2 CAMERA-Centre for the Analysis of Motion, Entertainment Research and Applications, University of Bath, Bath, UK; 3 Department of Bioengineering, Stanford University, Stanford, CA USA; 4 Department of Mechanical Engineering, Universitat Politècnica de Catalunya, Barcelona, Spain; 5 KIHU Finnish Institute of High Performance Sport, Jyväskylä, Finland

**Keywords:** Biomedical engineering, Computational biophysics, Biological physics

## Abstract

The current body of sprinting biomechanics literature together with the front-side mechanics coaching framework provide various technique recommendations for improving performance. However, few studies have attempted to systematically explore technique modifications from a performance enhancement perspective. The aims of this investigation were therefore to explore how hypothetical technique modifications affect accelerative sprinting performance and assess whether the hypothetical modifications support the front-side mechanics coaching framework. A three-dimensional musculoskeletal model scaled to an international male sprinter was used in combination with direct collocation optimal control to perform (data-tracking and predictive) simulations of the preliminary steps of accelerative sprinting. The predictive simulations differed in the net joint moments that were left ‘free’ to change. It was found that the ‘knee-free’ and ‘knee-hip-free’ simulations resulted in the greatest performance improvements (13.8% and 21.9%, respectively), due to a greater knee flexor moment around touchdown (e.g., 141.2 vs. 70.5 Nm) and a delayed and greater knee extensor moment during stance (e.g., 188.5 vs. 137.5 Nm). Lastly, the predictive simulations which led to the greatest improvements were also found to not exhibit clear and noticeable front-side mechanics technique, thus the underpinning principles of the coaching framework may not be the only key aspect governing accelerative sprinting.

## Introduction

Accelerative sprinting has attracted substantial research interest from a biomechanical perspective given its broad significance towards success within sporting contexts^1–7^. The studies carried out to date have predominantly focused their efforts on understanding ground reaction force and impulse production, as an athlete’s change in linear horizontal momentum is mainly governed by the net horizontal impulse they are able to generate during the stance phase. Thus, to improve accelerative sprinting performance, an athlete must generate greater horizontal net impulse, either through increasing the propulsive impulse, reducing the braking impulse, or by doing both. The generation of such forces is also dependent on the athlete’s technique prior to and during the stance phase, and therefore the relationship between the causes and consequences of sprinting motion needs to be thoroughly investigated.

One strategy, supported by current literature, is to reduce the braking impulse by generating high hip extension and knee flexion angular velocities during the terminal swing phase, through net hip extensor and knee flexor moments^8,9^. This is believed to reduce the horizontal foot velocity and centre of mass (CoM)-foot touchdown distance, and in turn reduce the braking impulse^3,8,10^. It is worthwhile noting that the complete removal of the braking phase may not be optimal, as this would potentially remove the benefits of the stretch–shortening cycle linked with the major ankle-spanning muscles^11,12^. Nevertheless, to date, no intervention studies have been carried out to ascertain whether this or alternative recommendations in general lead to performance enhancements. The lack of intervention studies is perhaps due to the unwillingness of athletes and their coaches to participate in such a study given its time consuming nature, and the potential negative impact it may have on their performance. A predictive computer simulation and modelling approach is instead very well-suited to performing such research, as this type of approach permits hypothetical ‘what if’ scenarios to be explored without the fear of jeopardising an athlete’s performance level^13,14^.

Another strategy is to maximise the propulsive impulse by ensuring that the thigh segment of the stance limb extends at a high velocity^10,15^. This is also linked to the strategy to minimise the braking impulse, as Mann and Murphy^16^ suggest that the net hip extensor moment generated during the beginning of the stance phase is crucial for this strategy. The work by Schache et al.^6^ provides evidence to support the claim made by Mann and Murphy^16^, as they found that there was a strong association between the angular impulse of the net hip extensor moment and forward acceleration whilst sprinting. Furthermore, the same study also identified a strong association between the angular impulse of the net ankle plantarflexor moment and forward acceleration. In a separate study, Bezodis et al.^2^ identified that amongst a group of three international calibre sprinters, the most distinguishing feature separating their performance levels during the first stance phase of accelerative sprinting was the magnitude and timing of the net knee extensor moment, which was greater and earlier in the highest performing sprinter. Furthermore, in accelerated sprinting the trunk is forwardly inclined, in comparison to maximum velocity^17^, and in such a configuration it is possible that the net knee extensor moment is favoured. It is interesting to note that the hip generates a net extensor moment for the portion of the stance phase which mainly involves braking, while the ankle and knee produce net plantarflexor and extensor moments, respectively, that span the braking portion and large parts of the propulsive portion. It is therefore possible that in accelerative sprinting, the net extensor and plantarflexor moments produced by the knee and ankle, respectively, may be of greater benefit to propulsive impulse generation than the hip.

**Figure 1:**
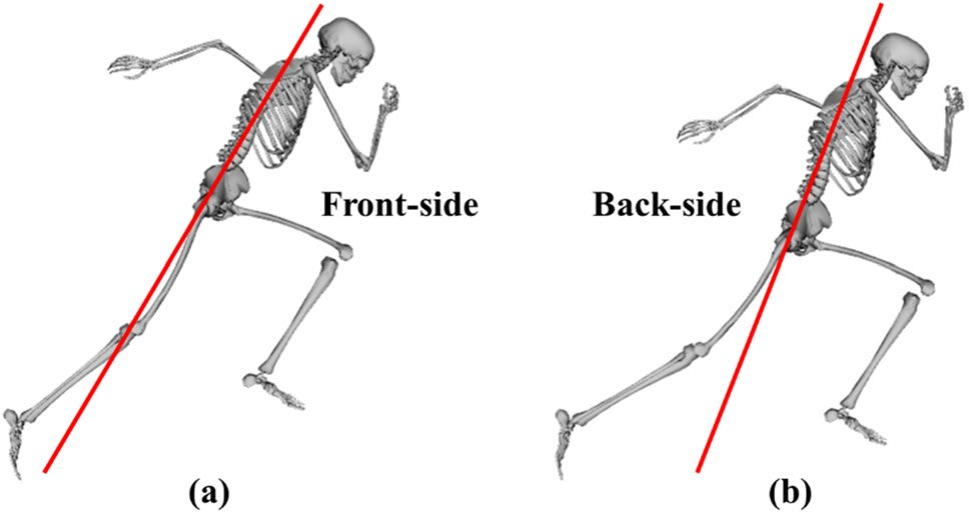
Front-side (**a**) and back-side (**b**) mechanics schematic during the second step stance phase of a maximal effort sprint. The solid red line drawn parallel to the torso indicates when the thigh segment is in front of the body and performing front-side mechanics or behind the body and performing back-side mechanics. Images generated using OpenSim 3.3 software (https://opensim.stanford.edu).

Many sport specific skills have techniques and movement patterns that coaches aim for their athletes to achieve based upon a coaching framework. The skill of sprinting is no different, and arguably the most well-known sprinting coaching framework is known as front-side mechanics, as proposed by Mann and Murphy^16^. The front-side mechanics coaching framework is based on the notion that the thigh segment should not extend past an imaginary line drawn parallel to the trunk when viewed from the sagittal plane (Fig. 1), and this is recommended from the onset of sprinting (independent of sprinting phase). In fact, when the thigh segment extends past this line, Mann and Murphy^16^ state that athletes sprint with back-side mechanics, which they deem as inefficient because an ineffective magnitude of ground reaction force can be produced during back-side mechanics and it subsequently leads to a longer stance phase duration. To achieve front-side mechanics, or prevent back-side mechanics, Mann and Murphy^16^ recommend that athletes should produce a net hip flexor moment during the latter 75% of the stance phase. A further potential benefit of executing this technique is that it should permit a greater ‘high knee’ position to be achieved during the swing phase, which would subsequently enable the thigh segment to be accelerated through a greater range of motion in preparation for the next stance phase and lead to greater ground reaction force production.

To date, however, the coaching framework proposed by Mann and Murphy^16^ has received limited attention from the scientific community, with only a limited number of studies focusing on it^18,19^. The recent study by Clark et al.^19^ provided evidence to support the claims of the framework, as they identified a moderate positive relationship between thigh angular range of motion and sprinting performance. Conversely, the findings in the study by Haugen et al.^18^ contradicted the proposed coaching framework, as they identified greater thigh extension at take-off to be associated with greater accelerative sprinting performance (i.e., sprinting with back-side mechanics was associated with greater performance). The findings from both studies should be interpreted with care based upon the athletes which participated in each study. In the study by Clark et al.^19^, the participants were from nonhomogeneous sporting backgrounds with markedly different sprinting abilities which could have potentially over inflated the relationship found. Whilst the participants in the study by Haugen et al.^18^ were sub-elite male sprinters (mean ± standard deviation 100 m PB: 10.86 ± 0.22 s), and Mann and Murphy^16^ suggest that back-side mechanics is only avoided by elite level sprinters, which is what sets them apart from the rest of their competitors. It is therefore still plausible that the front-side mechanics coaching framework is correct, however, further studies are needed to address this. Furthermore, there is the possibility that a different technical framework, yet to be uncovered, enables improved performance. For example, consider the high jump techniques prior to the advent of the Fosbury flop technique.

Predictive computer simulation and modelling approaches provide the platform for which to optimise the technique of a sporting movement, such as accelerative sprinting, and the usage of such an approach may help to provide further insights into the benefits of the front-side mechanics framework and demonstrate its merits from a scientific standpoint, which are currently lacking within the scientific literature. Nevertheless, despite the opportunities offered by such approaches, they have scarcely been applied to investigate sprinting, particularly from a technique and performance enhancement perspective. This was mainly due to the challenges in creating both a valid model and a robust simulation framework to realistically investigate such a demanding activity. In this study we tackled this challenge and performed predictive simulations of accelerative sprinting to investigate how modifications in technique affected performance, using a previously validated framework^20^. The secondary aim of this investigation was to provide insights into the front-side mechanics coaching framework, and to investigate whether the technique modifications coincided with improvements in performance as explained by the front-side mechanics coaching framework.

## Methods

### Musculoskeletal model

**Figure 2:**
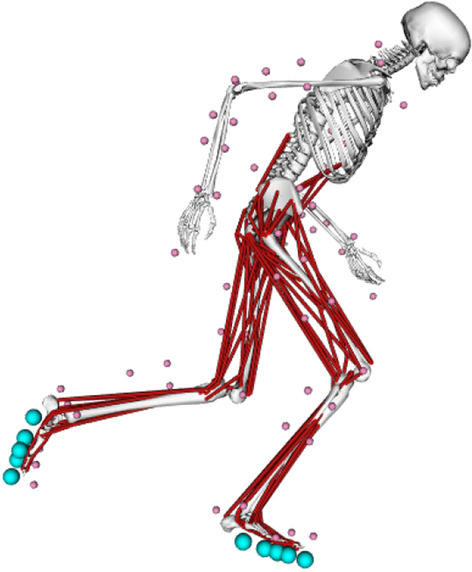
The three-dimensional musculoskeletal model used in this study (adapted from Hamner, et al.^26^). The human skeleton was modelled with 20 rigid segments: a pelvis, trunk (torso plus head), right and left lower-limbs (thigh, shank, upper rearfoot, lower rearfoot and forefoot) and upper-limbs (upper-arm, lateral forearm, medial forearm and hand). The DOFs of model were as follows: × 6 pelvis-to-ground, × 7 per lower-limb (× 3 hip, × 1 knee, × 1 ankle, × 1 subtalar and × 1 MTP), × 3 back, and × 7 per upper-limb (× 3 shoulder, × 2 elbow and × 2 wrist). The virtual model markers and smooth Hunt–Crossley contact spheres are denoted by pink and turquoise spheres, respectively. This same model was also used to perform the inverse kinematics and dynamics analyses to determine the kinematics and kinetics experimental tracking data. Imaged generated using OpenSim 3.3 software (https://opensim.stanford.edu).

The three-dimensional full-body musculoskeletal model described in Haralabidis et al.^20^ was used to perform the simulations in this study (Fig. 2). The human skeleton was modelled with 20 rigid segments and 37 degrees-of-freedom (DOFs). The lower-limb and trunk DOFs were actuated by 92 muscle–tendon units (MTUs) together with 17 reserve actuators. Each MTU was modelled as a three-element Hill-type model, with the contraction and activation dynamics of De Groote et al.^21^ and Zajac^22^, respectively. The lengths, velocities, and moment arms of the MTUs were described using differentiable and continuous polynomial functions^23^. The upper-limb DOFs were actuated by 14 joint actuators. The passive properties of sprinting spikes and the structures surrounding the forefoot were modelled by including a linear rotational spring at each of the metatarsophalangeal (MTP) DOFs. Foot–ground interaction was modelled by attaching four and two smooth Hunt–Crossley contact spheres^24^ to both of the model’s lower rearfoot and forefoot segments, respectively. The position of each contact sphere, and the stiffness and damping coefficients common to all the contact spheres were set to the values determined from a previous data-tracking simulation that tracked multiple trials simultaneously^20^. The aerodynamic drag force was also modelled by using the approach outlined by Samozino et al.^25^, and it was applied at the model’s CoM expressed in the local reference frame of the pelvis segment.

### Optimal control problem formulation and discretisation

The simulations were formulated as optimal control problems. Each optimal control problem was converted to a discrete nonlinear programming problem (NLP) by using direct collocation, which involves parameterising both the state and control variables. Specifically, the time horizon of the simulations was discretised across 150 equally spaced mesh intervals, with each mesh interval been further discretised with 4 points, by using a *flipped* Legendre–Gauss–Radau (LGR) direct collocation method^27^. The number of mesh intervals used in this study was greater than we had used in our previous data-tracking simulations of sprinting study^20^ due to simulating multiple stance phases. The state variables within each mesh interval were parameterised with third-order Lagrange polynomials. The control variables were parameterised at the beginning of a mesh interval and assumed to be piecewise constant throughout a mesh interval. In the following subsections, the model’s state and control variables are defined together with a description of how the musculoskeletal model’s dynamics were handled within the simulations, and these were common amongst all the simulations. The unique aspects (e.g., performance criterion and additional constraints) to the data-tracking and predictive simulations are also outlined.

### Dynamics formulation

The generalised coordinates ${\varvec{q}}$ and velocities ${\varvec{v}}$ of the musculoskeletal model, and the normalised tendon forces ${\widetilde{{\varvec{F}}}}_{{\varvec{T}}}$ and activations ${\varvec{a}}$ of the MTUs were selected as the state variables (${\varvec{x}}= {\left[{\varvec{q}}\,\boldsymbol{ }{\varvec{v}}\,\boldsymbol{ }{\widetilde{{\varvec{F}}}}_{{\varvec{T}}}\, {\varvec{a}}\right]}^{T}$). The upper-limb joint actuators ${{\varvec{u}}}_{{\varvec{U}}{\varvec{L}}}$, reserve actuators ${{\varvec{u}}}_{{\varvec{R}}{\varvec{E}}{\varvec{S}}}$, time derivatives of the generalised velocities ${{\varvec{u}}}_{\dot{{\varvec{v}}}}$, normalised tendon forces ${{\varvec{u}}}_{{\dot{\widetilde{{\varvec{F}}}}}_{{\varvec{T}}}}$ and activations ${{\varvec{u}}}_{\dot{{\varvec{a}}}}$, and ground reaction forces ${{\varvec{u}}}_{{\varvec{G}}{\varvec{R}}{\varvec{F}}}$ were selected as the control variables (${\varvec{u}}=\boldsymbol{ }{\left[\boldsymbol{ }{{\varvec{u}}}_{{\varvec{U}}{\varvec{L}}}\,\boldsymbol{ }{{\varvec{u}}}_{{\varvec{R}}{\varvec{E}}{\varvec{S}}}\,\boldsymbol{ }{{\varvec{u}}}_{\dot{{\varvec{v}}}}\,\boldsymbol{ }{{\varvec{u}}}_{{\dot{\widetilde{{\varvec{F}}}}}_{{\varvec{T}}}}\boldsymbol{ }{{\varvec{u}}}_{\dot{{\varvec{a}}}}\,\boldsymbol{ }{{\varvec{u}}}_{{\varvec{G}}{\varvec{R}}{\varvec{F}}}\right]}^{T}$). The chosen state and control variables enabled the differential equations underpinning the dynamics of the musculoskeletal model (skeletal, contraction and activation) to be represented in an implicit, as opposed to explicit, first-order state-space formulation^28,29^. The implicit dynamics formulation resulted in enforcing the musculoskeletal model’s first-order dynamics with simple differential constraints, and algebraic path constraints were included to enforce the underlying differential equations of the musculoskeletal model (Table 1). The first-order dynamics constraints were enforced at the LGR points within a mesh interval, whilst all the algebraic path constraints were enforced at the beginning of each mesh interval. Continuity constraints for the state variables between the ending and beginning of the mesh intervals were also included. Equality path constraints were included to ensure the ground reaction forces from both the control variable and the foot–ground contact model matched at an optimal solution.

**Table 1:** Overview of the musculoskeletal model’s constraints.

Dynamics constraints	$$\begin{array}{c}\frac{d{\varvec{q}}}{dt}=v \end{array}$$	(1.1)
	$$\begin{array}{c}\frac{d{\varvec{v}}}{dt}={{\varvec{u}}}_{\dot{{\varvec{v}}}} \end{array}$$	(1.2)
	$$\begin{array}{c}\frac{d{\widetilde{{\varvec{F}}}}_{{\varvec{T}}}}{dt}={{\varvec{u}}}_{{\dot{\widetilde{{\varvec{F}}}}}_{{\varvec{T}}}} \end{array}$$	(1.3)
	$$\begin{array}{c}\frac{d{\varvec{a}}}{dt}={{\varvec{u}}}_{\dot{{\varvec{a}}}} \end{array}$$	(1.4)
Path constraints	$$\begin{array}{c}M\left({\varvec{q}}\right)\cdot {{\varvec{u}}}_{\dot{{\varvec{v}}}}+C\left({\varvec{q}},{\varvec{v}}\right)+G\left({\varvec{q}}\right)-{{{\varvec{J}}}_{{\varvec{E}}{\varvec{x}}{\varvec{t}}}}^{T}\cdot Ext\left({{\varvec{u}}}_{{\varvec{G}}{\varvec{R}}{\varvec{F}}},{\varvec{A}}{\varvec{i}}{\varvec{r}}{\varvec{D}}{\varvec{r}}{\varvec{a}}{\varvec{g}}\right)-\left[\begin{array}{c}0\\{\varvec{\tau}}\end{array}\right]=0 \end{array}$$	(1.5)
	$$\begin{array}{c}{\widetilde{{\varvec{F}}}}_{{\varvec{T}}}-\mathrm{cos}\left({\varvec{\theta}}\left({\varvec{q}},{\widetilde{{\varvec{F}}}}_{{\varvec{T}}}\right)\right)\cdot \left({{\varvec{F}}}_{{\varvec{C}}{\varvec{E}}}\left({\varvec{q}},{\varvec{v}},{\widetilde{{\varvec{F}}}}_{{\varvec{T}}},\boldsymbol{ }{\varvec{a}}\right)+{{\varvec{F}}}_{{\varvec{P}}{\varvec{E}}}\left({\varvec{q}},{\widetilde{{\varvec{F}}}}_{{\varvec{T}}}\right)\right)=0 \end{array}$$	(1.6)
	$$\begin{array}{c}0\le {{\varvec{u}}}_{\dot{{\varvec{a}}}}+\frac{{\varvec{a}}}{{\tau }_{d}}; {\tau }_{d}=60\,ms\end{array}$$	(1.7)
	$$\begin{array}{c}{{\varvec{u}}}_{\dot{{\varvec{a}}}}+\frac{{\varvec{a}}}{{\tau }_{a}}\le \frac{1}{{\tau }_{a}}; {\tau }_{a}=15\,ms \end{array}$$	(1.8)
	$$\begin{array}{c}{{\varvec{H-C}}}_{{\varvec{G}}{\varvec{R}}{\varvec{F}}}\left({\varvec{q}},{\varvec{v}}\right)-{{\varvec{u}}}_{{\varvec{G}}{\varvec{R}}{\varvec{F}}}=0 \end{array}$$	(1.9)
Continuity constraints	$$\begin{array}{c}{{\varvec{x}}}_{i}^{END}-{{\varvec{x}}}_{i+1}^{1}=0 \end{array}$$	(1.10)

Skeletal dynamics were enforced as per equation $(1.5)$, where ${\varvec{M}}\left({\varvec{q}}\right)$ is the mass matrix, ${\varvec{C}}\left({\varvec{q}},{\varvec{v}}\right)$ is the vector of centrifugal forces, ${\varvec{G}}\left({\varvec{q}}\right)$ is the vector of gravitational forces, ${{{\varvec{J}}}_{{\varvec{E}}{\varvec{x}}{\varvec{t}}}}^{T}$ is the transpose of the external forces Jacobian matrix, ${\varvec{E}}{\varvec{x}}{\varvec{t}}\left({{\varvec{u}}}_{{\varvec{G}}{\varvec{R}}{\varvec{F}}},{\varvec{A}}{\varvec{i}}{\varvec{r}}{\varvec{D}}{\varvec{r}}{\varvec{a}}{\varvec{g}}\right)$ is the vector of external ground reaction and air drag forces and ${\varvec{\tau}}$ is the vector of net joint moments (consisting of the moments generated by the MTUs, upper-limb and reserve actuators, and springs attached to the MTP DOFs). Contraction dynamics were imposed using the Hill model equilibrium condition $(1.6)$, where the normalised tendon force must equal the projected sum of the normalised contractile ${{\varvec{F}}}_{{\varvec{C}}{\varvec{E}}}\left({\varvec{q}},{\varvec{v}},{\widetilde{{\varvec{F}}}}_{{\varvec{T}}},\boldsymbol{ }{\varvec{a}}\right)$ and passive ${{\varvec{F}}}_{{\varvec{P}}{\varvec{E}}}\left({\varvec{q}},{\widetilde{{\varvec{F}}}}_{{\varvec{T}}}\right)$ muscle forces. Activation dynamics were enforced using the inequality constraint equations $(1.7-1.8)$, and they were derived from the original differential equation describing activation dynamics^30^. Equation $(1.9)$ was imposed to ensure consistency between the ground reaction forces calculated from the contact model ${{\varvec{H-C}}}_{{\varvec{GRF}}}\left({\varvec{q}},{\varvec{v}}\right)$ and the controls ${{\varvec{u}}}_{{\varvec{G}}{\varvec{R}}{\varvec{F}}}$.

### Data-tracking simulation

To perform the data-tracking simulation it was first necessary to perform an empirical data collection. A thorough overview of the data collection procedures can be found in Haralabidis et al.^20^, and thus only a summary is given here. Three-dimensional marker trajectories (250 Hz, Qualisys AB, Sweden) and ground reaction forces (2000 Hz, Kistler, Switzerland) were collected from an international-level male sprinter (age: 24 years; height: 1.79 m; mass: 72.2 kg; 100 m PB: 10.33 s; 200 m PB: 20.27 s) as he performed two successful maximal effort sprints over three distances (0–10, 0–30 and 0–60 m). The participant provided written informed consent to take part in the data collection. The data collection protocol was approved by the University of Bath’s Research Ethics Approval Committee for Health (EP 17/18 238). All methods were carried out in accordance with relevant guidelines and regulations. For the purposes of this study, the data collected from between the touchdown of the second step (right foot) to the take-off of the third step (left foot) during the first 0–10 m trial (early acceleration phase) was extracted for further use. The extracted data together with the model described above were then used to perform inverse kinematics (global pelvis and relative joint angles) and dynamics (net joint moments) analyses within OpenSim (version 3.3, Stanford University, CA, USA)^31^. The kinematics and ground reaction forces were filtered using a fourth-order low-pass Butterworth filter with a 20 Hz cut-off frequency prior to performing the inverse dynamics analysis. B-splines were also fitted to the filtered kinematics data to enable velocities and accelerations to be determined. The splined kinematics, filtered ground reaction forces and net joint moments served as the experimental data to be tracked within the data-tracking simulation.

For the data-tracking simulation, the objective function ${J}_{TrackSim}$ consisted of three terms: a tracking term ${J}_{Tracking}$ that minimised the errors between experimental and simulated kinematics, ground reaction forces and net joint moments (excluding the net MTP moments), an effort term ${J}_{Effort}$ that minimised the activations, and a control variables term ${J}_{Control}$ that minimised the reserve actuators control variables and those control variables introduced to permit the use of an implicit dynamics formulation (${{\varvec{u}}}_{\dot{{\varvec{v}}}}\,\boldsymbol{ }{{\varvec{u}}}_{{\dot{\widetilde{{\varvec{F}}}}}_{{\varvec{T}}}}\,\boldsymbol{ }{{\varvec{u}}}_{\dot{{\varvec{a}}}}$):

1 $${J}_{TrackSim}= {J}_{Tracking}+{J}_{Effort}+{J}_{Control}$$

2 $${J}_{Tracking}={w}_{1}\sum_{j=1}^{37}\int_{0}^{{t}_{f}}{\left(\frac{{q}_{j}^{EXP}-{q}_{j}^{SIM}}{range\left({q}_{j}^{EXP}\right)}\right)}^{2}dt+{w}_{2}\sum_{n=1}^{6}\int_{0}^{{t}_{f}}{\left(\frac{{GRF}_{n}^{EXP}-{u}_{{GRF}_{n}}^{SIM}}{range\left({GRF}_{n}^{EXP}\right)}\right)}^{2}dt+ {w}_{3}\sum_{k=1}^{29}\int_{0}^{{t}_{f}}{\left(\frac{{\tau }_{k}^{EXP}-{\tau }_{k}^{SIM}}{range\left({\tau }_{k}^{EXP}\right)}\right)}^{2}dt$$

3 $${J}_{Effort}={w}_{4}\sum_{i=1}^{92}\int _{0}^{{t}_{f}}\left(\frac{{F}_{i}^{max}{a}_{i}^{S{IM}^{2}}}{\sum_{i=1}^{92}{F}_{i}^{max}}\right)dt$$

4 $${J}_{Control}= {w}_{5}\sum_{m=1}^{17}\int _{0}^{{t}_{f}}{\left(\frac{{u}_{{res}_{m}}^{SIM}}{bound\left({u}_{{res}_{m}}^{SIM}\right)}\right)}^{2}dt+{w}_{6}\sum_{j=1}^{37}\int _{0}^{{t}_{f}}{\left(\frac{{u}_{{\dot{v}}_{j}}^{SIM}}{range\left({\ddot{q}}_{j}^{EXP}\right)}\right)}^{2}dt+ {w}_{7}\sum_{i=1}^{92}\int_{0}^{{t}_{f}}{\left(\frac{{u}_{{\dot{\widetilde{F}}}_{{T}_{i}}}^{SIM}}{bound\left({u}_{{\dot{\widetilde{F}}}_{{T}_{i}}}^{SIM}\right)}\right)}^{2}dt+ {w}_{7}\sum_{i=1}^{92}\int_{0}^{{t}_{f}}{\left(\frac{{u}_{{\dot{a}}_{i}}^{SIM}}{bound\left({u}_{{\dot{a}}_{i}}^{SIM}\right)}\right)}^{2}dt$$

where the superscripts $EXP$ and $SIM$ denote the experimental and simulated variables, respectively, ${t}_{f}$ (0.436 s) denotes the duration of the extracted data from the trial being tracked, ${\tau }_{k}$ are the net joint moments, ${F}_{i}^{max}$ are the MTU maximal isometric force parameters, and ${w}_{i}$ are the weights of the tracking and minimisation terms. The values of the weights were set using a heuristic approach based on the importance placed on the term being tracked or minimised (${\varvec{w}}=$[0.1 0.05 0.01 0.01 0.001 0.0001 0.1]), as currently there is no unanimous agreement within the relevant biomechanical literature for how they should be selected. The net MTP moments from the inverse dynamics analysis were not tracked due to experimental challenges related to the centre of pressure. The calculated differences between the experimental and simulated variables within ${J}_{Tracking}$ (excluding the anterior–posterior pelvis translation difference), and the time derivatives of the generalised velocities control variables were normalised by 10% of each variables experimental range (determined from the trial being tracked). The anterior–posterior pelvis translation difference was normalised by 0.01 m to ensure it was tracked accurately. The remainder of the variables within ${J}_{Control}$ were normalised by their respective upper bounds.

The initial guess alongside the lower and upper bounds of the state and control variables were set using the experimental data of the trial being tracked where possible. The splined experimental kinematics were used to initialise ${\varvec{q}}$, ${\varvec{v}}$ and ${{\varvec{u}}}_{\dot{{\varvec{v}}}}$, and their bounds were set to be 25% more than and less than the maximum and minimum of the values, respectively, obtained from the splined kinematics. The initial guess for the ${{\varvec{u}}}_{{\varvec{U}}{\varvec{L}}}$ was set using the results from the inverse dynamics analysis. The ${{\varvec{u}}}_{{\varvec{R}}{\varvec{E}}{\varvec{S}}}$ and ${{\varvec{u}}}_{{\varvec{G}}{\varvec{R}}{\varvec{F}}}$ were initialised as zero, with bounds of ± 10 Nm (40 Nm MTP DOFs) and ± 2500 N, respectively. The same initial guess and bounds as Falisse et al.^28^ were used for ${\widetilde{{\varvec{F}}}}_{{\varvec{T}}}$, ${\varvec{a}}$, ${{\varvec{u}}}_{{\dot{\widetilde{{\varvec{F}}}}}_{{\varvec{T}}}}$ and ${{\varvec{u}}}_{\dot{{\varvec{a}}}}$.

### Predictive simulations

In previous predictive simulation studies of sporting tasks, for instance jumping^32^ and sprinting^33^, the objective has been to predict novel movements without considering technique changes. However, the primary objective of the predictive simulations carried out in this investigation was to explore how modifications in sprinting technique can lead to improvements in performance during the early acceleration phase. We explored modifications to sprinting technique from a net joint moments paradigm as they are the drivers for movement, as described previously, and we focused on the net ankle, knee, and hip flexor–extensor moments. Specifically, the predictive simulations were performed by enabling either individual or combinations of the listed net joint moments to freely vary whilst the remaining net joint moments within the predictive simulations aimed to coincide with those determined from the data-tracking simulation. The net joint moments which we were not attempting to modify were tracked to permit subsequent inferences to changes in performance to be attributed to the net joint moments which we gave the freedom to change. Seven predictive simulations were performed in total. Three of the predictive simulations enabled the net ankle, knee, and hip flexor–extensor moments to independently vary freely (A-free, K-free and H-free), another three predictive simulations enabled combinations of two of the net joint moments to independently vary freely (A-K-free, A-H-free and K-H-free) and the final simulation enabled all three of the net joint moments to independently vary freely (A-K-H-free). The time horizon duration was also treated as a variable to be determined from each of the predictive simulations. The following constraints were imposed for each predictive simulation:

5 $${\left[{{\varvec{q}}}^{\mathrm{1,0}} {{\varvec{v}}}^{\mathrm{1,0}}\right]}^{T}= {\left[{{\varvec{q}}}_{Track}^{\mathrm{1,0}}\, {{\varvec{v}}}_{Track}^{\mathrm{1,0}}\right]}^{T}$$

6 $${q}_{{Pelvis}_{x}}^{END,0}= {q}_{{Pelvis}_{{x}_{Track}}}^{END,0}$$

7 $$-10^\circ \le {{\varvec{q}}}_{JointAngles}^{END,0}-{{\varvec{q}}}_{{JointAngles}_{Track}}^{END,0}\le 10^\circ$$

the first constraint $(5)$ was imposed to ensure the state variables of the musculoskeletal model’s multibody dynamics at the beginning of the simulation matched with those determined from the data-tracking simulation; the second constraint $(6)$ ensured that the anterior–posterior displacement of the musculoskeletal model’s pelvis segment at the end of the simulation matched with the value obtained from the data-tracking simulation; and the third constraint $(7)$ was imposed to ensure that the relative joint angles of the musculoskeletal model at the end of the simulation fell within ± 10° of those obtained from the data-tracking simulation.

For each of the predictive simulations, the cost function ${J}_{PredSim}$ consisted of four terms: a duration of time horizon minimisation term ${J}_{Time}$, a tracking term ${J}_{Tracking}$ that minimised the differences between certain data-tracking simulation and predictive simulation net joint moments, an effort term ${J}_{Effort}$ that minimised the activations, and a control variables term ${J}_{Control}$ that minimised the reserve actuators control variables and those control variables introduced to permit the implicit dynamics formulation (${{\varvec{u}}}_{\dot{{\varvec{v}}}}\,\boldsymbol{ }{{\varvec{u}}}_{{\dot{\widetilde{{\varvec{F}}}}}_{{\varvec{T}}}}\,\boldsymbol{ }{{\varvec{u}}}_{\dot{{\varvec{a}}}}$):

8 $${J}_{PredSim}={J}_{Time}+{J}_{Tracking}+{J}_{Effort}+{J}_{Control}$$

9 $${J}_{Time}={W}_{1}{t}_{f}^{Pred}$$

10 $${J}_{Tracking}={W}_{2}\sum_{a=1}^{31}{w}^{\tau }\int _{0}^{{t}_{f}^{Pred}}{\left(\frac{{\tau }_{a}^{Track}-{\tau }_{a}^{Pred}}{range\left({\tau }_{a}^{Track}\right)}\right)}^{2}dt$$

11 $${J}_{Effort}={W}_{3}\sum_{i=1}^{92}\int _{0}^{{t}_{f}^{Pred}}\left(\frac{{F}_{i}^{max}{a}_{i}^{S{IM}^{2}}}{\sum_{i=1}^{92}{F}_{i}^{max}}\right)dt$$

12 $${J}_{Control}= {W}_{4}\sum_{m=1}^{17}\int _{0}^{{t}_{f}^{Pred}}{\left(\frac{{u}_{{res}_{m}}^{SIM}}{bound\left({u}_{{res}_{m}}^{SIM}\right)}\right)}^{2}dt+{W}_{5}\sum_{j=1}^{37}\int_{0}^{{t}_{f}^{Pred}}{\left(\frac{{u}_{{\dot{v}}_{j}}^{SIM}}{range\left({\ddot{q}}_{j}^{EXP}\right)}\right)}^{2}dt+ {W}_{6}\sum_{i=1}^{92}\int_{0}^{{t}_{f}^{Pred}}{\left(\frac{{u}_{{\dot{\widetilde{F}}}_{{T}_{i}}}^{SIM}}{bound\left({u}_{{\dot{\widetilde{F}}}_{{T}_{i}}}^{SIM}\right)}\right)}^{2}dt+ {W}_{6}\sum_{i=1}^{92}\int_{0}^{{t}_{f}^{Pred}}{\left(\frac{{u}_{{\dot{a}}_{i}}^{SIM}}{bound\left({u}_{{\dot{a}}_{i}}^{SIM}\right)}\right)}^{2}dt$$

where ${t}_{f}^{Pred}$ represents the duration of the time horizon to be determined, ${\tau }_{a}^{Track}$ and ${\tau }_{a}^{Pred}$ denote the $a$’th tracking (obtained from the tracking simulation) and predictive simulation net joint moment, ${W}_{i}$ are the weights of the cost function terms being minimised or tracked (${\varvec{W}}=$[50 0.1 0.01 1 0.0001 0.1]), and ${{\varvec{w}}}^{\tau }$ is a binary vector with ones at the indices of the net joint moments that were tracked and zeros at the other indices (those given the freedom to vary). The data-tracking simulation and predictive simulation variable differences within ${J}_{Tracking}$ were normalised by 2% of their respective range as determined from the data-tracking simulation. This was done to ensure that the net joint moments which we did not want to freely change were still tracked reasonably accurately. For example, the threshold selected coincided with a permissible error of 5 Nm assuming a 250 Nm range. The variables within ${J}_{Control}$ were normalised as per the data-tracking simulation.

The initial guess for the state and control variables for each simulation were set to the values determined from the data-tracking simulation. The lower and upper bounds of the state and control variables were identical to those used in the data-tracking simulation. The lower bound of ${t}_{f}^{Pred}$ was set to 5% less than the original value of ${t}_{f}$ used in the data-tracking simulation (0.414 s), whilst the upper bound was kept at the original value of ${t}_{f}$.

### Optimal control problem solution approach

The data-tracking and predictive simulations were formulated in MATLAB (2017b; MathWorks Inc., Natick, MA, USA) using CasADi^34^, and solved using IPOPT^35^ with an adaptive barrier parameter strategy and NLP convergence tolerance of 10^–3^. The variables for each NLP were scaled to lay on the interval [− 1, 1] and the constraints were scaled to improve the convergence rate and numerical conditioning as per recommendations of Betts^36^.

### Outcome measures

The data-tracking simulation was evaluated by calculating the root mean squared difference (RMSD) between the tracked experimental data and simulated data. The performance of the data-tracking and predictive simulations was quantified using average horizontal external power^37^ and was calculated as the rate of change in kinetic energy between the start and end of each simulation. The horizontal CoM velocity at the end of each simulation and the time horizon were also extracted as performance outcomes. The horizontal impulses (net, propulsive and braking) were calculated across each stance phase using trapezoidal quadrature. We also extracted touchdown and take-off kinematics-based technique variables based on existing literature (horizontal foot touchdown velocity and horizontal CoM-foot touchdown distance) and front-side mechanics principles (take-off hip extension, knee flexion, thigh extension and trunk orientation). Lastly, to further foster comparison between the data-tracking and predictive simulations from a net joint moments perspective, the peak flexor and extensor net joint moments during stance were extracted.

## Results

### Data-tracking simulation

The kinematics and kinetics from the data-tracking simulation were found to closely match the corresponding experimental data (Supplementary material Figs. S1 to S4). The average RMSDs were less than 1° and 0.3 cm for the global pelvis angles and translations, respectively, and less than 1° for the relative joint angles. The right and left stance phase ground reaction force components were tracked with an RMSD of 0.050 and 0.054 BW (anterior–posterior), 0.034 and 0.033 BW (vertical) and 0.003 and 0.009 BW (medial–lateral). The average RMSDs of the tracked net joint moments and the net lower-limb joint moments were 16.3 and 17.5 Nm, respectively. These results demonstrate that the modelling and simulation framework used was a sufficient representation of reality and therefore suitable for performing the proposed predictive simulations.

### Predictive simulations

#### Performance outcomes

**Figure 3:**
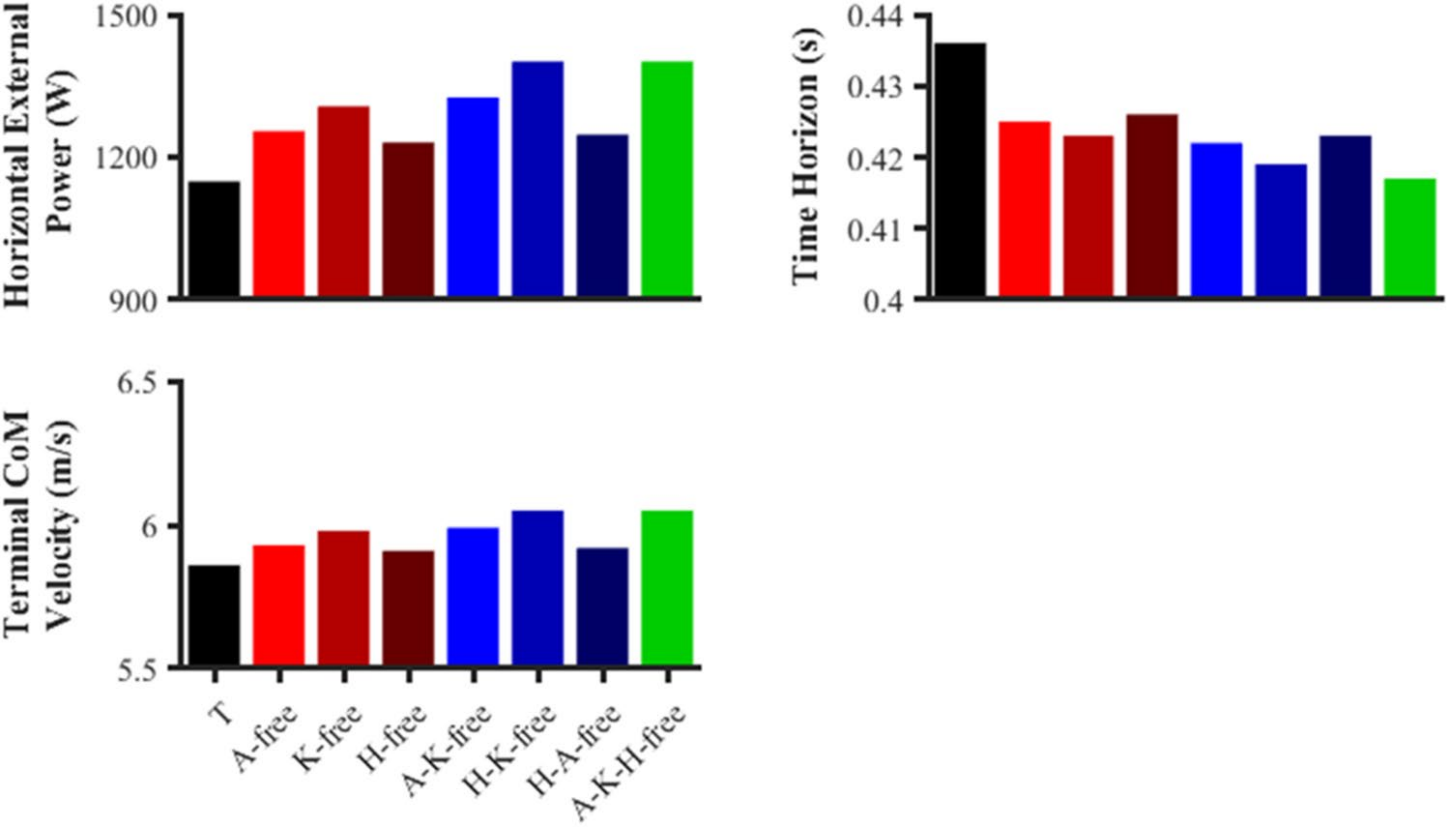
Metrics to quantify the sprint performance in the data-tracking (T) and predictive simulations (A-free, K-free, H-free, A-K-free, H-K-free, H-A-free, A-K-H-free).

Each of the predictive simulations performed resulted in improvements to overall performance as indicated by the average horizontal external power (Fig. 3). The greatest improvement in performance was found for A-K-H-free (22.0%; 1401.2 vs. 1148.7 W), and this simulation also led to the greatest improvements in terminal horizontal CoM velocity (3.2%; 6.05 vs. 5.86 m/s) and time horizon duration (− 4.4%; 0.417 vs. 0. 436 s).

#### Horizontal impulses

**Figure 4:**
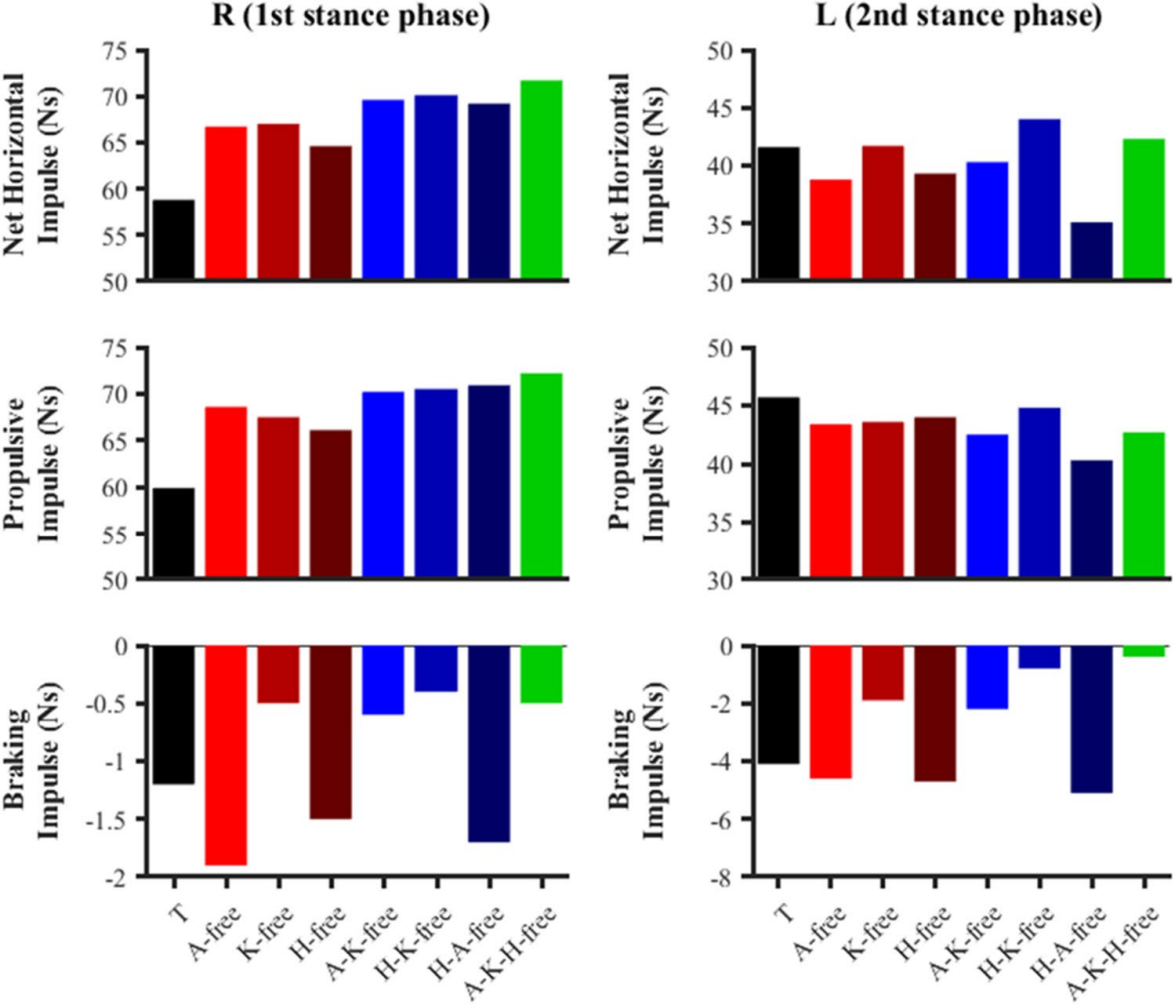
Right (R) and left (L) stance phase anterior–posterior impulses for the data-tracking (T) and predictive simulations (A-free, K-free, H-free, A-K-free, H-K-free, H-A-free, A-K-H-free).

All the predictive simulations were found to generate greater net horizontal impulse during the right foot stance phase compared to the data-tracking simulation (average of 68.4 vs. 58.8 Ns). For the left foot stance phase, the net horizontal impulse generated was lower in several of the predictive simulations (A-free, H-free, A-K-free, H-A-free) compared to the data-tracking simulation (Fig. 4), but not for the predictive simulation that led to the largest performance improvements (A-K-H-free). Despite the lower net horizontal impulse of those simulations, the net horizontal impulse surplus they generated during the right foot stance phase was still sufficient to produce an overall improvement in terminal horizontal CoM velocity (Fig. 3).

Similar to the above, the predictive simulations were found to generate greater propulsive impulse during the right foot stance phase compared to the data-tracking simulation (average of 69.4 vs. 59.9 Ns). However, all the predictive simulations were found to produce less propulsive impulse during the left foot stance phase (average of 43.0 vs. 45.7 Ns) (Fig. 4). There was not a clear trend in the simulation results for the braking impulse, although the predictive simulations which led to the greatest improvements in performance (A-K-H-free and H-K-free) produced lower braking impulses (average of left: − 0.5, average of right: − 0.6 Ns). Time histories of the ground reaction force components can be found within the supplementary material for completeness (Fig. S5).

#### Timings

**Figure 5:**
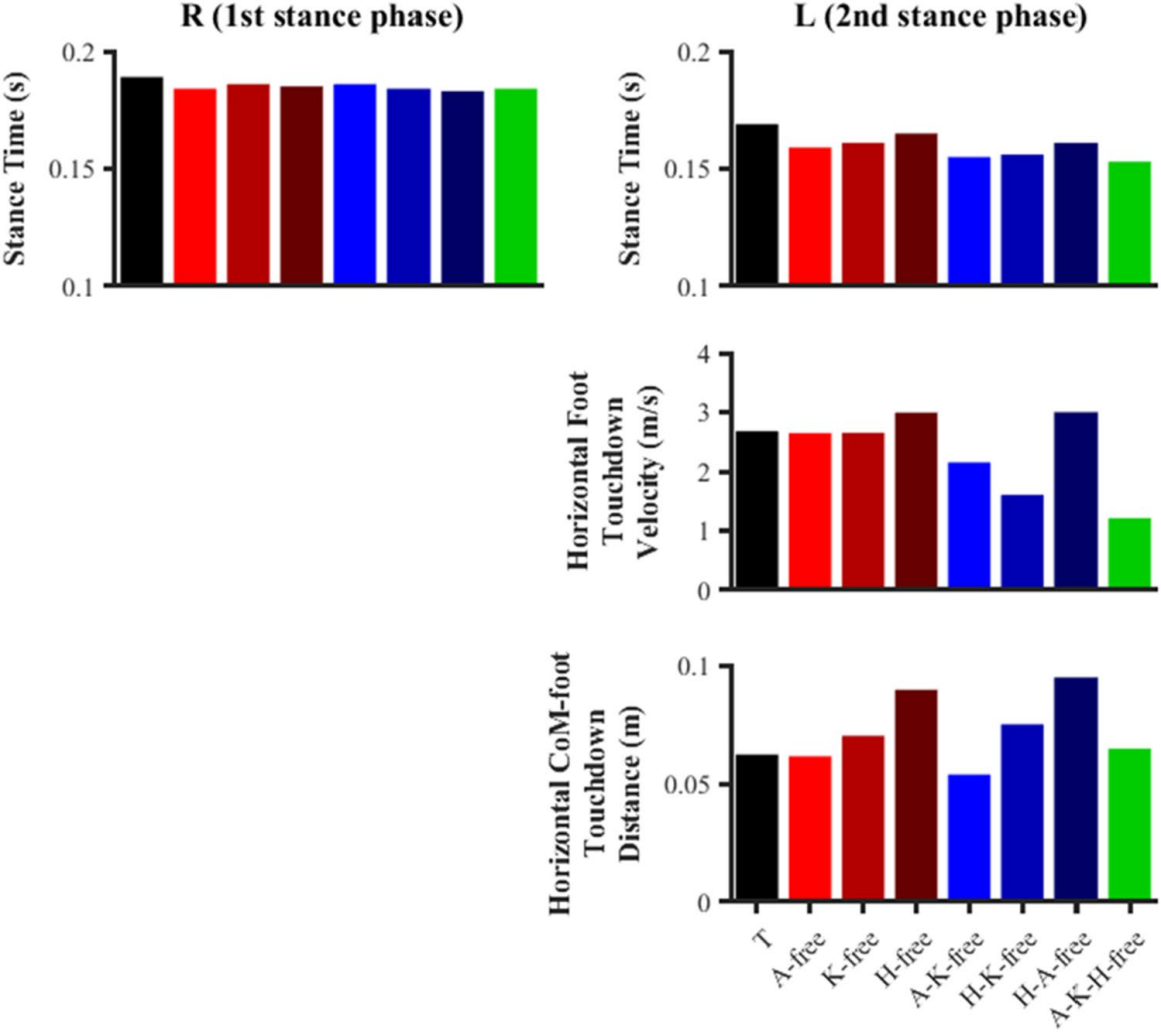
Right (R) and left (L) touchdown kinematics and stance phase durations for the data-tracking (T) and predictive simulations (A-free, K-free, H-free, A-K-free, H-K-free, H-A-free, A-K-H-free). A positive horizontal CoM-foot touchdown distance indicates that the foot was ahead of the CoM.

Very minor differences in the duration of the right foot stance phases were observed between the data-tracking and predictive simulations (average of 0.185 vs. 0.189 s), however, potentially more meaningful reductions were found for the left foot stance phases (average of 0.159 vs. 0.169 s) (Fig. 5).

#### Kinematics

The horizontal foot touchdown velocity (0.98 m/s) and the horizontal CoM-foot touchdown distance (0.09 m) of the right foot stance phase were identical for all simulations performed, as the state variables of the musculoskeletal model’s multibody dynamics for the predictive simulations were constrained to match those determined from the data-tracking simulation. A trend was observed for the horizontal foot touchdown velocity of the left foot stance phase to be lower as performance was found to increase (Fig. 5). The lowest horizontal foot touchdown velocity of the left foot stance phase was found to be 1.21 m/s for the A-K-H-free predictive simulation. There was no noticeable trend for the horizontal CoM-foot touchdown distance of the left foot stance phase, with values ranging between 0.054 and 0.095 m.

**Figure 6:**
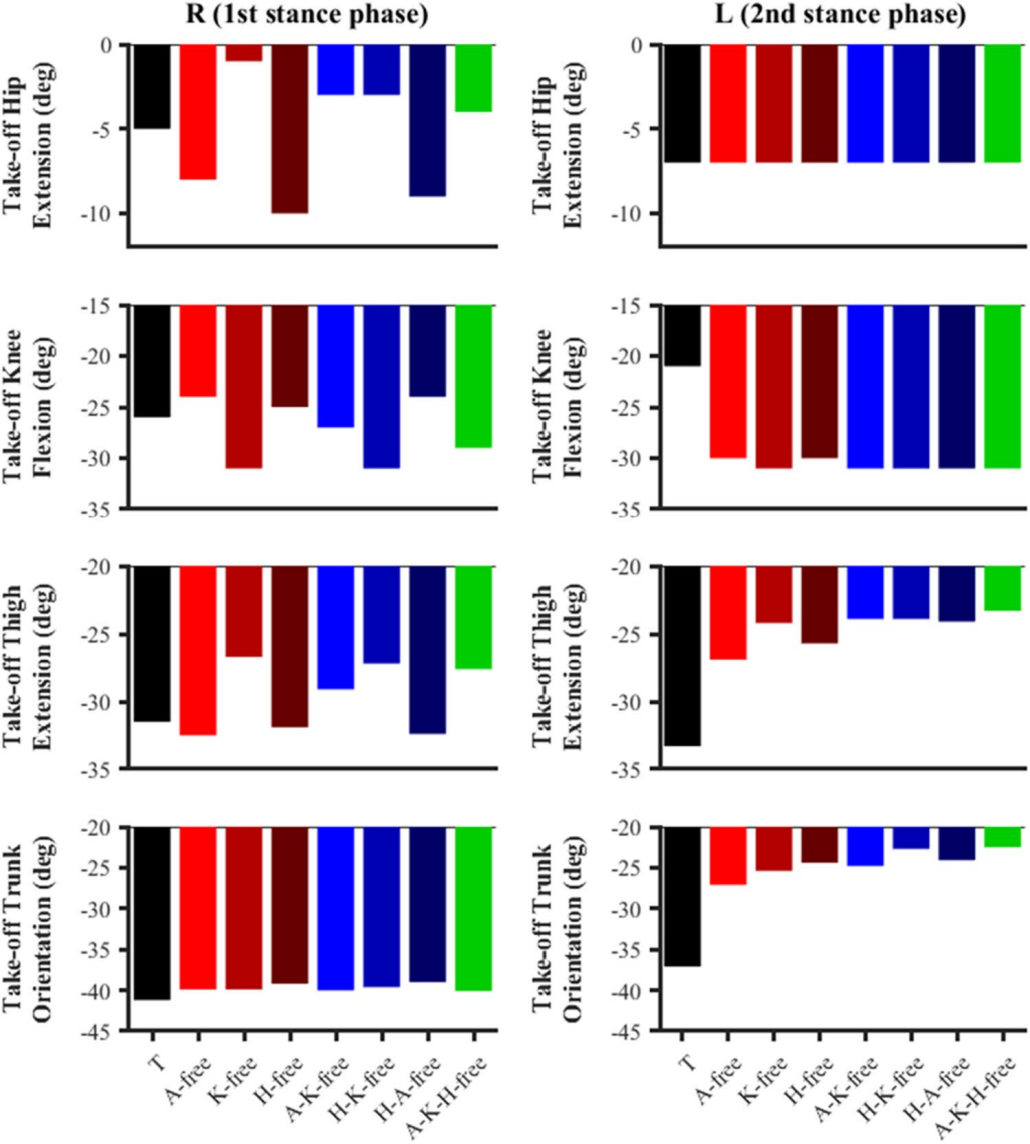
Right (R) and left (L) hip extension, knee flexion, thigh extension and trunk orientation angles at take-off for the data-tracking (T) and predictive simulations (A-free, K-free, H-free, A-K-free, H-K-free, H-A-free, A-K-H-free). Full knee extension = 0° and knee flexion is negative; thigh aligned vertically with pelvis corresponds to hip flexion–extension = 0°, and hip flexion is positive and hip extension is negative. Thigh and trunk segment angles are defined relative to the vertical axis of the global coordinate system. Negative thigh and trunk segment angles (clockwise rotations) correspond to extension and forward inclination, respectively. These are consistent with the model’s definitions.

The ‘knee-free’ predictive simulations showed lower hip extension (− 1° to − 4°) and higher knee flexion (− 27° to − 31°) angles at take-off for the right foot stance phase compared to the data-tracking simulation (Fig. 6). For the left foot stance phase, all simulations were found to exhibit a hip extension take-off angle of -7°, whilst showing a greater knee flexion angle at take-off compared to the data-tracking simulation (− 31° vs. − 21°).

#### Front-side vs back-side mechanics

**Figure 7:**
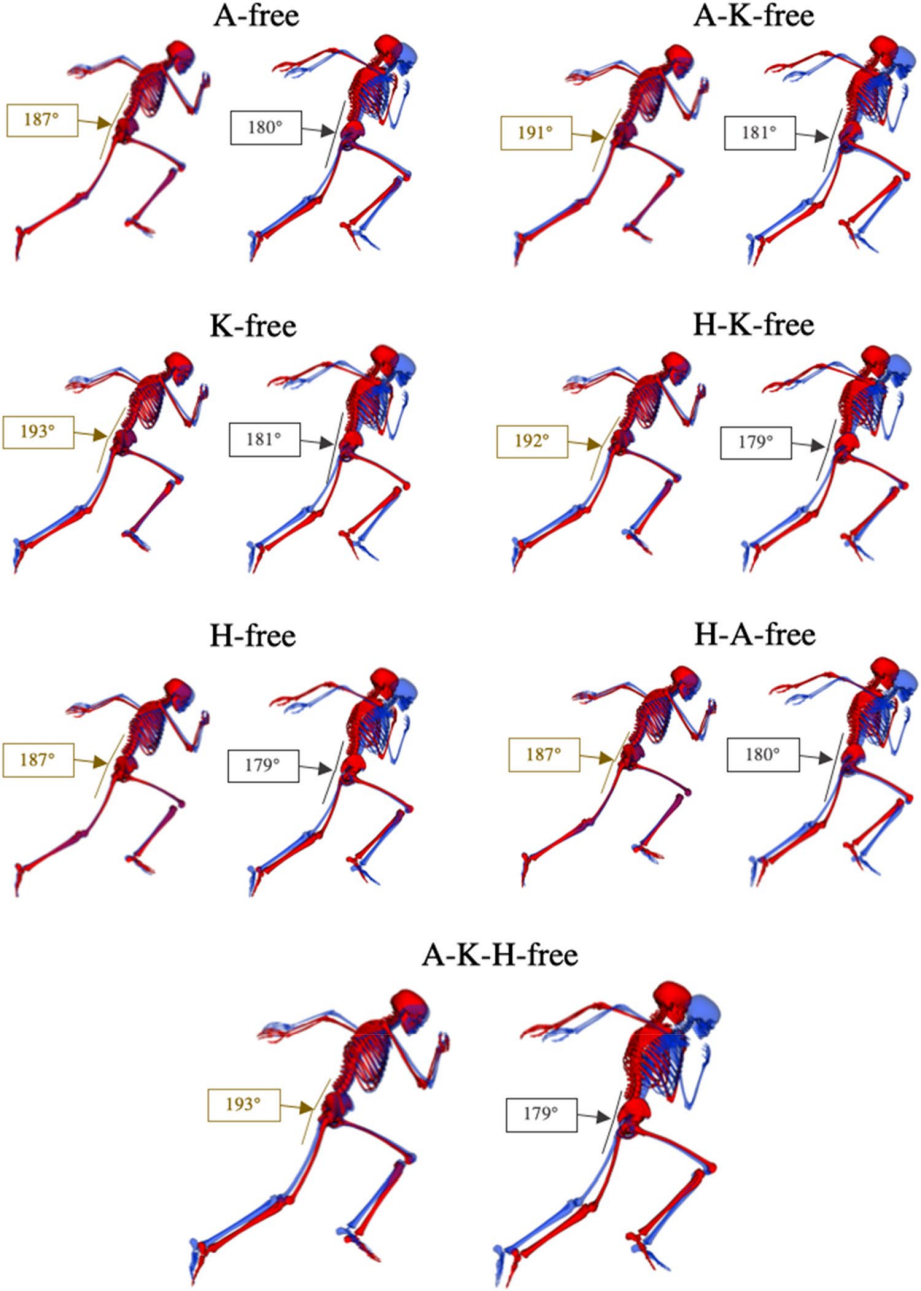
Right and left foot take-off configurations for the data-tracking (blue model) and predictive simulations (red model). The angles included are between the trunk and thigh segments for the predictive simulations, with angles greater than 180° reflecting front-side mechanics. Images generated using OpenSim 3.3 software (https://opensim.stanford.edu).

Overall, at take-off for the right foot stance phase, both the data-tracking and the predictive simulations showed an absolute trunk to thigh angle greater than 180°, which is, theoretically, indicative of the adoption of front-side mechanics. In the ‘knee-free’ simulations this angle slightly increased (from 1° to 3°) with respect to the data-tracking simulation, as the thigh orientation decreased whilst the trunk position remained almost unaltered (Fig. 6). However, during the left foot stance phase, although the data-tracking simulation still showed front-side mechanics features (trunk to thigh angle = 184°), all predictive simulations showed a decrease at least greater than 3° from that value. This is due to the thigh and trunk orientation take-off angles concomitantly decreasing (Fig. 6), which leads to cancelling out the front-side mechanics pattern. This can be seen in the take-off configurations presented in Fig. 7, where the trunk is always in a more upright position with respect to the data-tracking simulation.

**Figure 8:**
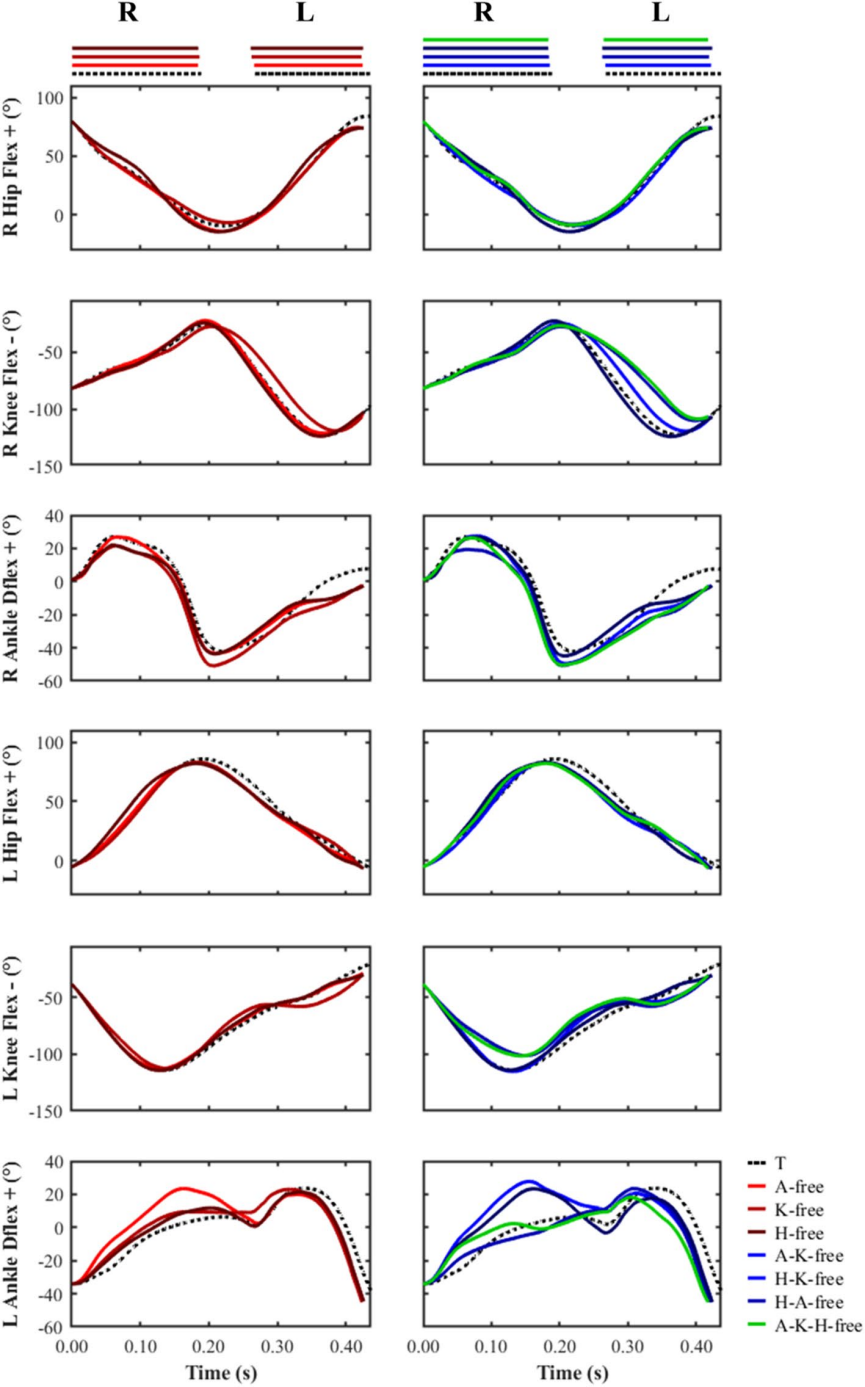
Right and left ankle dorsiflexion-plantarflexion angles, and knee and hip flexion–extension angles from right foot touchdown to left foot take-off for the data-tracking (T) and predictive simulations (A-free, K-free, H-free, A-K-free, H-K-free, H-A-free, A-K-H-free). The horizontal bars at the top of the figure indicate periods of right (R) and left (L) stance. The outputs from the simulations were not plotted on the same set of axes to avoid interpretability and visualisation issues.

For the swing phase following the right foot stance phase, all the predictive simulations were found to exhibit reduced left hip flexion compared to the data-tracking simulation (82° vs. 86°) (Fig. 8). For the ‘knee-free’ predictive simulations, we also identified a knee flexion reduction for the right limb during the swing phase (− 109° vs. − 122°). The interested reader can find the time histories of the global pelvis translations and angles in the supplementary material (Fig. S6).

#### Net joint moments

**Figure 9:**
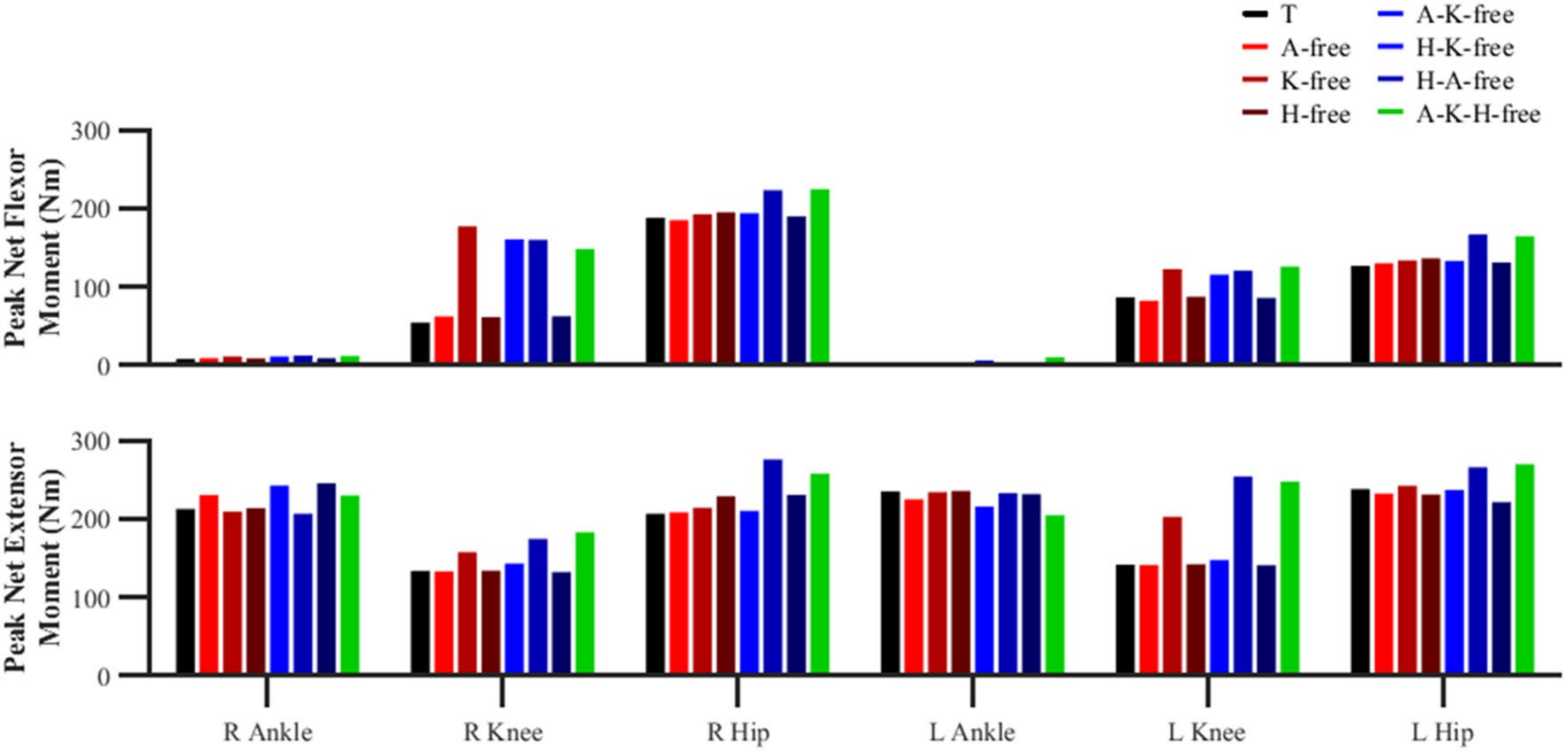
Right (R) and left (L) peak ankle dorsiflexor and plantarflexor net moments, and peak knee and hip net flexor and extensor moments during the right and left stance phases for the data-tracking (T) and predictive simulations (A-free, K-free, H-free, A-K-free, H-K-free, H-A-free, A-K-H-free).

**Figure 10:**
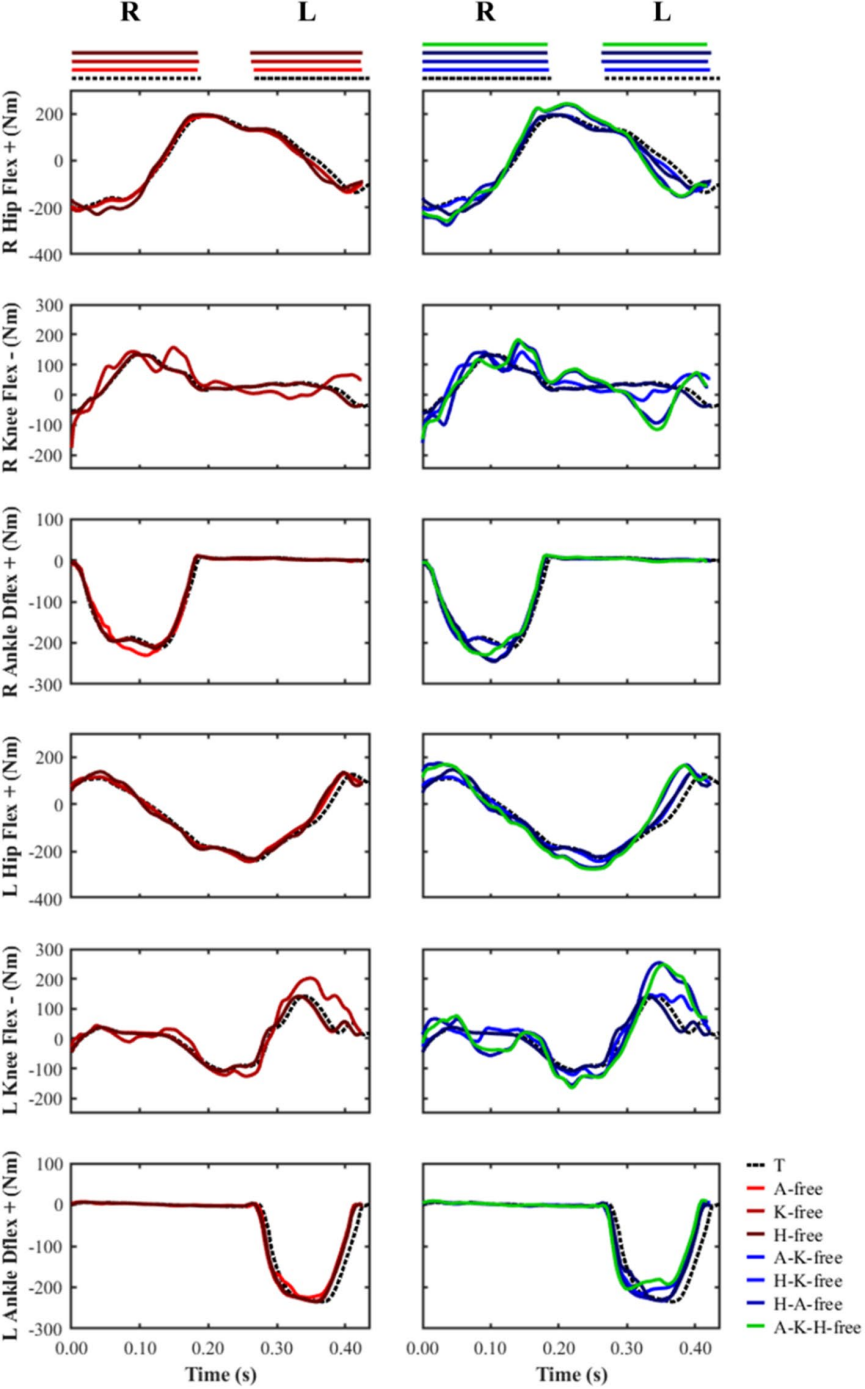
Right and left net ankle dorsiflexor-plantarflexor moments, and knee and hip flexor–extensor moments from right foot touchdown to left foot take-off for the data-tracking (T) and predictive simulations (A-free, K-free, H-free, A-K-free, H-K-free, H-A-free, A-K-H-free). The horizontal bars at the top of the figure indicate the periods of right (R) and left (L) stance. The outputs from the simulations were not plotted on the same set of axes to avoid interpretability and visualisation issues.

The most discernible differences in the peak net flexor and extensor moments were observed for the knee and hip (Fig. 9). The ‘knee-free’ simulations were found to generate a greater peak net flexor (average 100.3%; 141.2 vs. 70.5 Nm) and extensor moment (average 37.1%; 188.5 vs. 137.5 Nm) across both right and left foot stance phases compared to the data-tracking simulation. In addition, the stance phase peak net knee flexor moment was found to occur at the beginning of stance for all simulations, whilst the peak net knee extensor moment occurred ~ 40% later in stance for the ‘knee-free’ predictive simulations compared to the data-tracking simulation (Fig. 10). Furthermore, for the same set of predictive simulations we also observed a substantial net knee flexor moment for the limb in the swing phase in comparison to the data-tracking simulation (greatest difference: − 115.3 vs. 39.9 Nm), which occurred at ~ 50% of the stance limb’s stance phase.

The H-K-free and A-K-H-free predictive simulations were found to generate greater peak net flexor (average 23.6%; 194.5 vs. 157.3 Nm) and extensor (average 20.2%; 266.9 vs. 222.9 Nm) moments across both stance phases in comparison to the data-tracking simulation. The reversal from net hip extensor moment to net hip flexor moment was also identified as occurring earlier in the stance phase in these predictive simulations compared to the data-tracking simulation (~ 30% vs. ~ 35%), together with an increased rate of change (Fig. 10). Lastly, the peak net hip flexor moment for this subset of predictive simulations was greater than the data-tracking simulation during both the right (greatest difference: 242.1 vs. 194.6 Nm) and left (greatest difference: 168.8 vs. 115.0 Nm) lower-limb flight phases.

## Discussion

The primary purpose of this investigation was to identify how modifications in technique influence performance during the preliminary steps of accelerative sprinting. This was achieved by using a predictive simulation and modelling approach where the lower-limb (ankle, knee, and hip) flexor–extensor net joint moments were given the freedom to be modified both individually and in combination. We found that the predictive simulations which enabled the net knee moments to vary (K-free) resulted in the greatest improvements in performance. More specifically, the K-free and K-H-free simulations resulted in a 13.8% and 21.9% increase of average horizontal external power, and the A-K-H-free simulation, was found to generate the highest average horizontal external power (22.0%). Videos of the kinematics from the data-tracking and predictive simulations, in addition to the data files, can be accessed online at https://doi.org/10.6084/m9.figshare.14988051.

The secondary aim of this study was to evaluate whether the improvements in performance shown by the predictive simulations were in line with the front-side mechanics coaching framework, for which there is currently a limited amount of peer-reviewed scientific literature. The subset of ‘hip-free’ simulations showed partial evidence of the recommended front-side mechanics technique, but only from a joint kinetics perspective. These simulations, during the late stance and swing phases, were characterised by an earlier and greater generation of net hip flexor moments. Surprisingly, however, these changes in net hip flexor moments translated into a reduced, rather than increased, hip flexion angle during the swing phase, which is not what the front-side mechanics framework advocates. Furthermore, considering the combined trunk and thigh orientation in the sagittal plane at take-off as the main criterion to assess the adoption of front side mechanics, all predictive simulations showed a reduction in this angle at take-off of the left foot stance phase and tended towards more neutral values (~ 180°) (Figs. 6, 7). Such results, therefore, suggest that the underpinning principles of front-side mechanics might not be the only key aspects governing accelerating sprinting, and it is perhaps more beneficial to avoid back-side mechanics, than fully embrace a front-side mechanics technique. This hypothesis is also reinforced by the fact that the predictive simulations which led to the greatest improvements in performance (i.e., ‘knee-free’) did not show clear evidences of front-side mechanics technique.

The changes in net joint moments timings and magnitude, which are linked to the greatest performance enhancement, were generated by the subset of simulations where the knee net moments were left free to vary. Amongst these simulations, a greater and earlier net knee flexor moment, prior to the initiation of the left foot stance phase, was generated (Fig. 10). The potential benefit of this aspect of technique is to reduce the magnitude of the braking impulse by driving the lower-limb backwards at touchdown and therefore reducing the horizontal touchdown velocity of the foot^3,10^. Indeed, especially for the subset of ‘knee-free’ simulations (A-K free, H-K free, and A-H-K free), the magnitude of the braking impulse decreased (Fig. 4), together with a reduction in the horizontal touchdown velocity of the foot (Fig. 5). The net hip extensor moment during the late swing phase has been suggested to perform a similar role as the net knee flexor moment^3,10^, and we observed this phenomenon in the H-free and H-K-free predictive simulations (Fig. 10). Greater reductions in the horizontal foot touchdown velocity (left foot stance phase) and the braking impulses were observed in the H-K-free predictive simulation, thus demonstrating the combined benefits of the actions of those net joint moments. The technique improvements from those net joint moments are potentially due to an enhanced coordination (from a timing and magnitude perspective) of the hamstring muscles, as they are responsible for both knee flexion and hip extension. Furthermore, Morin, et al.^38^ identified the significance of the hamstring muscles in the late swing phase in relation to ground reaction force production based on electromyography measurements. The computational modelling and simulation approach utilised in this study permitted exploring the behaviour of the hamstring muscles more thoroughly, however, it was deemed beyond the scope of this study. In further work we will perform a detailed analysis of the results at a muscle level to explore potential changes in the coordination of the hamstring muscles.

The above findings demonstrate that a key feature of improving acceleration performance is by minimising the braking impulses via the generation of greater net knee flexor and net hip extensor moments. However, previous experimental studies^1,5,7^ have found that improved early accelerative sprinting is based on an individual’s ability to generate greater propulsive impulses as opposed to reduced braking impulses, which tends to differentiate sprinters more in the latter stages of accelerative sprinting. A potential explanation for this discrepancy is due to the different study designs embraced. The studies by Colyer et al.^1^, Morin et al.^7^ and Nagahara et al.^5^ used a cross-sectional study approach, whilst the predictive simulations carried out in this study can almost be viewed as enabling a highly controlled intervention study, where individualised enhancement in performance was achieved via reducing the braking impulses. However, the results in this study are also dependent on the characteristics of the musculoskeletal model used, as the strategy for improving performance may have been different (i.e., increase propulsive impulses) due to differing organismic constraints (e.g., inertial or MTUs parameters)^39,40^, and similarly the conclusions with regards to front-side mechanics may also have been different as a consequence. Nevertheless, we believe that this is a particular strength of the approach adopted, as individualised technique recommendations to improve performance can be administered instead of group level recommendations which may or may not be beneficial for a particular athlete.

The ‘knee-free’ predictive simulations were found to increase the magnitude of the peak net knee extensor moment during the late stance phase in comparison to the data-tracking simulation. This finding is in line with a previous study^2^, which focused on the lower-limb net joint kinetics of three international calibre athletes during the first stance phase of accelerative sprinting. Specifically, Bezodis et al.^2^ identified that the most distinguishing feature of technique in relation to performance was the net knee extensor moment, as the highest performing athlete was found to generate a greater and earlier net knee extensor moment. The results from our simulations therefore provide further evidence to support the existing notion of the link between the behaviour of the net knee extensor moment and early accelerative sprinting performance. Interestingly, Schache et al.^6^ found that there was a strong association between the angular impulses of the net hip extensor and ankle plantarflexor moments and forward acceleration during accelerative sprinting. Interpreting our findings together with the work of Schache et al.^6^ and Bezodis et al.^2^ suggest that the net knee joint moments have a major performance benefitting role in early accelerative sprinting. However, the net ankle and hip joint moments are also fundamental to continue enabling an athlete to accelerate their CoM whilst sprinting.

A further important aspect of technique that we identified was the increase in magnitude and timing of the net hip flexor moment, particularly for the second stance phase, in the ‘hip-free’ simulations. This technique aspect has been suggested to be imperative for reversing the rotation of the hip (extension to flexion), and Mann and Murphy^16^ have stipulated that the highest calibre athletes generate a net hip flexor moment for the latter ~ 75% of the stance phase. More specifically, it is thought to prevent unnecessary extension at the hip and permits front-side mechanics dominance (e.g., greater hip flexion and higher knee lift). In the ‘hip-free’ predictive simulations we see the transition to earlier and greater net hip flexor moments when compared to the data-tracking simulation, with a net hip flexor moment for ~ 65% of the stance phase. Despite the changes in the hip joint moments coinciding with the front-side mechanics recommendation, we did not also observe increases in hip flexion during swing, instead reductions. This was a surprising result, as the work by Clark et al.^19^ found a positive linear relationship between hip flexion and sprinting velocity, thus, providing evidence in favour of this aspect of front-side mechanics. Further work is needed to clarify the significance of this aspect of front-side mechanics given the discrepancy in findings. Nevertheless, given that the greatest improvements in performance from our simulations were identified from the ‘knee-free’ simulations, it is possible that technique-based factors not covered by front-side mechanics were more influential to the modelled athlete’s sprinting performance.

It is possible that the kinematics-based criteria of front-side mechanics during the swing phase (i.e., increased hip flexion) did not emerge from the predictive simulations due to the margins of improvement in performance. For instance, the eight male finalists competing at the 2018 60 m World Athletics Championships achieved second and third stance phase durations between 0.153–0.193 and 0.120–153 s, respectively^41^. Meanwhile the lowest second and third stance phase durations for the predictive simulations were 0.183 and 0.153 s, respectively, and a previous study has reported a strong association between stance phase duration and accelerative sprinting performance^42^. Whilst these stance phase durations give credibility to the predictive simulation results, the lack of increased hip flexion may explain why they are higher than the upper bounds achieved by the finalists. This also coincides with the beliefs held by Mann and Murphy^16^, in which only the highest calibre athletes are able to perform with front-side mechanics. The lack of clear front-side mechanics dominance emerging from our predictive simulations may also be due to insufficient strength within the musculoskeletal model or the properties and geometry of the MTUs not been representative of an elite sprinter. For instance, the moment arms of the major knee extensor muscles have been shown to be greater in sprinters compared to non-sprinters^43^. Future work should therefore aim to disentangle the ability to perform front-side mechanics by potentially using a predictive computational modelling and simulation approach, as per this study, together with a subject-specific model informed by medical imaging (e.g., magnetic resonance imaging) to explore the confounding factors described.

At the instant of take-off, the data-tracking simulation already showed trunk to thigh angles (right take-off: 190°; left take-off: 184°), which reflected the adoption of front-side mechanics technique. The predictive simulations showed small and inconsistent changes in the trunk to thigh angle (± 3°) during the right foot take-off, and a consistent reduction in the trunk to thigh angle during the left foot take-off (Fig. 7). The changes in trunk and thigh angles are cancelling each other out from a front- (or back-) side mechanics perspective, and the net effect results in a neutral position (180° ± 1) where the thigh is in line with the theoretical trunk line showed in Fig. 1. Thus, we believe that this might be seen as a mechanism to prevent back-side mechanics from occurring at take-off. It is worthwhile to acknowledge that these results may have been obtained due to the modelling choices taken to actuate the trunk segment, which is actuated by a small subset of muscles together with reserve actuators and is thus a simplified representation of reality. Consequently, the trunk behaviour in the current study arguably represents the best-case scenario, and with a more detailed representation of the muscles spanning the pelvis-trunk joint perhaps it would not have been possible for the trunk to attain the changes in inclination necessary to prevent back-side mechanics. To date, one of the rare studies^18^ to explore the recommendations proposed by front-side mechanics at take-off found that the directions of the relationships between key variables did not coincide (e.g., greater thigh extension and running velocity). Nevertheless, the avoidance of back-side mechanics relies on the combined effect of the thigh and trunk segments, which Haugen et al.^18^ did not consider, and isolated analyses of each of the variables does not suffice. Furthermore, the cohort of athletes analysed within the same study did not feature elite sprinters, which may again potentially explain the discrepancies in the relationships between the technique variables and performance they obtained.

An interesting finding from the predictive simulations was that they tended to show a trend of increasing the net and propulsive impulses in the first stance phase compared to the data-tracking simulation, whilst the net and propulsive impulses of the second stance phase were less than those obtained in the data-tracking simulation. Considering that all the predictive simulations improved performance compared to the data-tracking simulation, a greater surplus of net impulse from the first stance phase had to be produced by the predictive simulations such that overall performance was still enhanced. We believe that this result can be attributed to the inequality constraint enforcing that the relative joint angles at the end of the predictive simulations were within ± 10° of those obtained at the end of the data-tracking simulation. This may have restricted the possibility of achieving optimal solutions featuring greater net and propulsive impulses for the second stance phase. We imposed the inequality constraint to ensure that the musculoskeletal model was posed such that it had the possibility of performing a subsequent step. Without imposing this type of constraint, the final solution obtained by the algorithm more closely resembled jumping/hopping/diving as opposed to accelerative sprinting, as we found in our previous work^44^. In the future, it would be interesting to simulate more than two stance phases to explore whether the imposed inequality constraint was the cause of this result.

In this study, we used a cost function that included a tracking element, which in our case was a subset of the entire set of net joint moments. This simulation approach was similar to the ones utilised by Meyer et al.^45^ and van den Bogert et al.^46^ for performing predictive simulations of walking and running, respectively. We opted to embrace such an approach to ensure we were subsequently able to infer changes in performance through systematic modifications to the net ankle, knee and hip flexor–extensor moments, and to ensure the predicted outputs were still representative of sprinting. However, we recognise this is a limitation of our study as assigning different weightings to the tracking (subset of the entire set of net joint moments) and performance (minimisation of the time horizon duration) terms would have led to different results. Interestingly, the weighting assigned to the minimisation of time horizon duration meant that it never reached its lower bound, and we believe that this ensured a balancing of the two terms in the cost function such that neither was too heavily minimised.

Previous studies have performed predictive simulations of sprinting to address a variety of research purposes^33,47,48^. However, to the authors’ knowledge this is the first study to perform predictive simulations of sprinting whilst using state-of-the-art computational modelling and simulation approaches (e.g., three-dimensional musculoskeletal model) for the purposes of exploring the interactions between technique and performance. In previous studies, model complexity and simulation duration were sacrificed likely due to the large computational cost incurred to solve an OCP together with the inefficient means of discretising the OCP. In this study we performed predictive simulations of sprinting by transcribing the OCP using direct collection, formulated the musculoskeletal dynamics implicitly and used algorithmic differentiation, and together they have been shown to increase computational efficiency and the ability of obtaining an optimal solution^21,32^. Furthermore, previous studies which have performed predictive simulations of walking, running, or sprinting^23,47^ have imposed a symmetry constraint at the end of their simulations to improve computational tractability. However, this is not appropriate in circumstances in which asymmetry between steps is likely, such as accelerative sprinting which was the form of locomotion studied in this investigation.

The findings from the current investigation are purely theoretical as they are based on the outputs from a predictive modelling and simulation framework, which must be acknowledged when drawing conclusions from our results. The framework used in this study was found to be a sufficient representation of reality, and it has also been previously shown in a separate study^20^, which provides confidence in the outputs. Arguably the biggest criticism of this study is the potential for an athlete to implement the key technique-based findings, as coaching instructions are typically provided from a kinematics perspective. However, we took a different approach, based on net joint moments modifications, as ultimately the net joint moments produced by the muscles are responsible for motion. Recently, Richards et al.^49^ utilised real-time feedback featuring targeted net joint moments patterns together with kinematics cues to successfully modify walking gait. Such developments in real-time feedback and biofeedback are promising solutions to explore the effectiveness of the recommendations proposed in the current study in the real world.

## Conclusion

The results from the predictive simulations highlighted that modifications in technique due to the timing and magnitude of the net knee flexor–extensor moments resulted in the greatest improvements to overall performance. The ‘hip-free’ predictive simulations led to an earlier net hip flexor moment during the stance phase, although this did not translate to greater hip flexion during the swing phase which the front-side mechanics coaching framework advocates. Interestingly, in all the predictive simulations we observed the tendency to avoid back-side mechanics at take-off. Taken together, the results from the current study suggest that the front-side mechanics recommendations during the swing phase are not necessarily the limiting aspect of improving performance, as the predictive simulations converged to differing techniques than proposed by front-side mechanics. It is worthwhile noting that the improvements in accelerative sprinting performance we found can be viewed as short-term technique modifications, instead of long-term adaptations via strength and conditioning programmes, as the properties of the model were kept constant between predictive simulations. Lastly, for coaches and sport scientists, the results suggest that cueing the actions of the knee and hip flexors and extensors as identified by the predictive simulations, particularly during the late swing (knee flexors and hip extensors) and stance (knee extensors and hip flexors) phases, could improve performance.

## Supplementary Information

Supplementary Information.

## Data Availability

The datasets generated and/or analysed during the current study are available at the publicly available Figshare repository titled: modifications to the net knee moments lead to the greatest improvements in accelerative sprinting performance: a predictive simulation study, with the following link: https://doi.org/10.6084/m9.figshare.14988051.
