# Supplementary material for: Modifications to the net knee moments lead to the greatest improvements in accelerative sprinting performance: a predictive simulation study
Source: Sci Rep. 2022 Sep 23;12:15908. doi: 10.1038/s41598-022-20023-y (PMC9508344; doi:10.1038/s41598-022-20023-y)
Supplement: Supplementary file 1 — Supplementary Information. [file 41598_2022_20023_MOESM1_ESM.pdf]

Modifications to the net knee moments lead to the greatest improvements in accelerative sprinting performance: a predictive simulation study

Supplementary material

Nicos Haralabidis*[1,2,3], Steffi L. Colyer[1,2], Gil Serrancolí[4], Aki I.T. Salo[5,1,2] & Dario Cazzola[1,2]

[1] Department for Health, University of Bath, UK

[2] CAMERA-Centre for the Analysis of Motion, Entertainment Research and Applications, University of Bath, UK

[3] Department of Bioengineering, Stanford University, Stanford, California, USA

[4] Department of Mechanical Engineering, Universitat Politècnica de Catalunya, Barcelona, Spain

[5] KIHU Finnish Institute of High Performance Sport, Jyväskylä, Finland

Corresponding Author:
Nicos Haralabidis*[1,2,3]
Claverton Down, Bath, BA2 7AY, UK
Email address: nicosharalabidis@gmail.com

Data-tracking simulation results

Time histories of the ankle dorsiflexion-plantarflexion angles and net moments, knee and hip flexion-extension angles and net moments, pelvis kinematics, and ground reaction forces for the data-tracking simulation together with the tracked experimental data are presented in Figures S1 to S4.

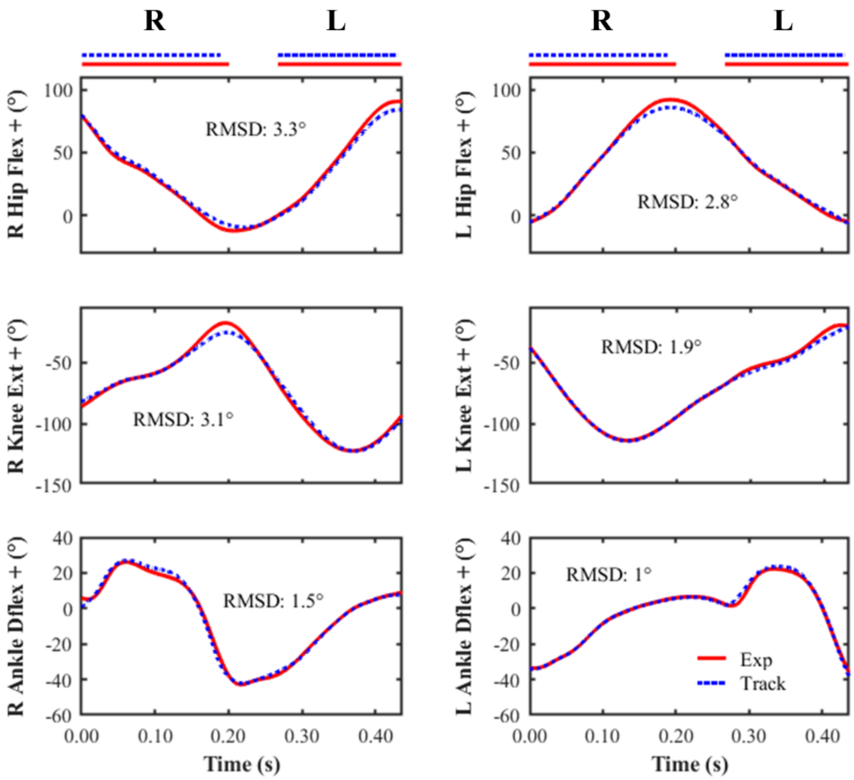

Figure S1 Right and left ankle dorsiflexion-plantarflexion angles, and knee and hip flexion-extension angles from right foot touchdown to left foot take-off. Experimental joint angles are denoted by solid red lines (EXP). Simulated joint angles are denoted by dashed blue lines (TRACK). The horizontal bars at the top of the figure indicate the periods of right (R) and left (L) foot stance.

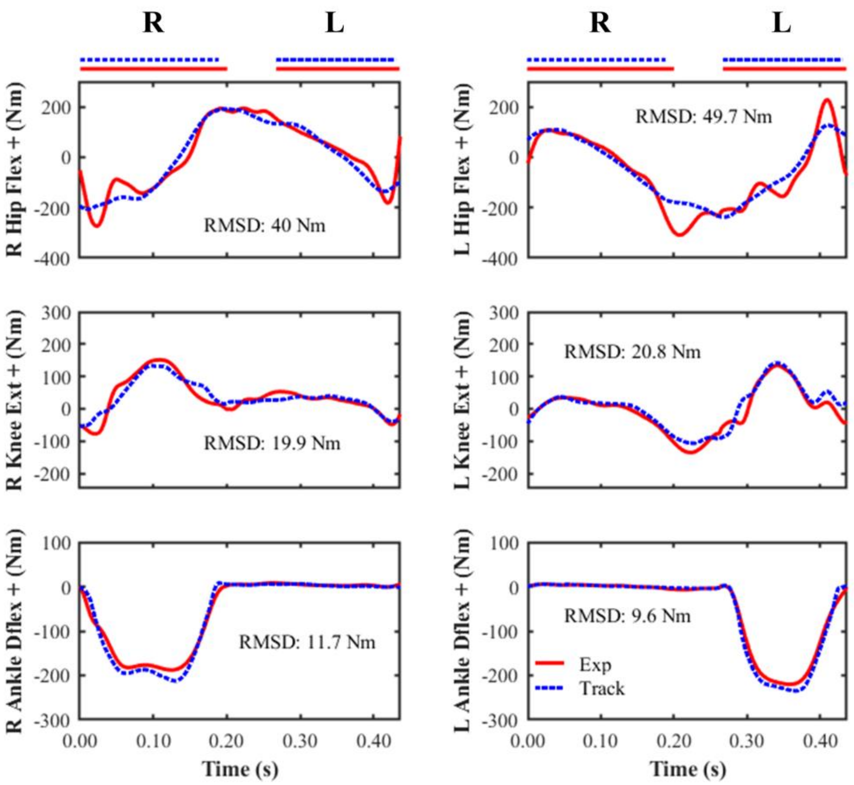

Figure S2 Right and left net ankle dorsiflexor-plantarflexor moments, and knee and hip flexor-extensor moments from right foot touchdown to left foot take-off. Experimental net joint moments are denoted by solid red lines (EXP). Simulated net joint moments are denoted by dashed blue lines (TRACK). The horizontal bars at the top of the figure indicate the periods of right (R) and left (L) foot stance.

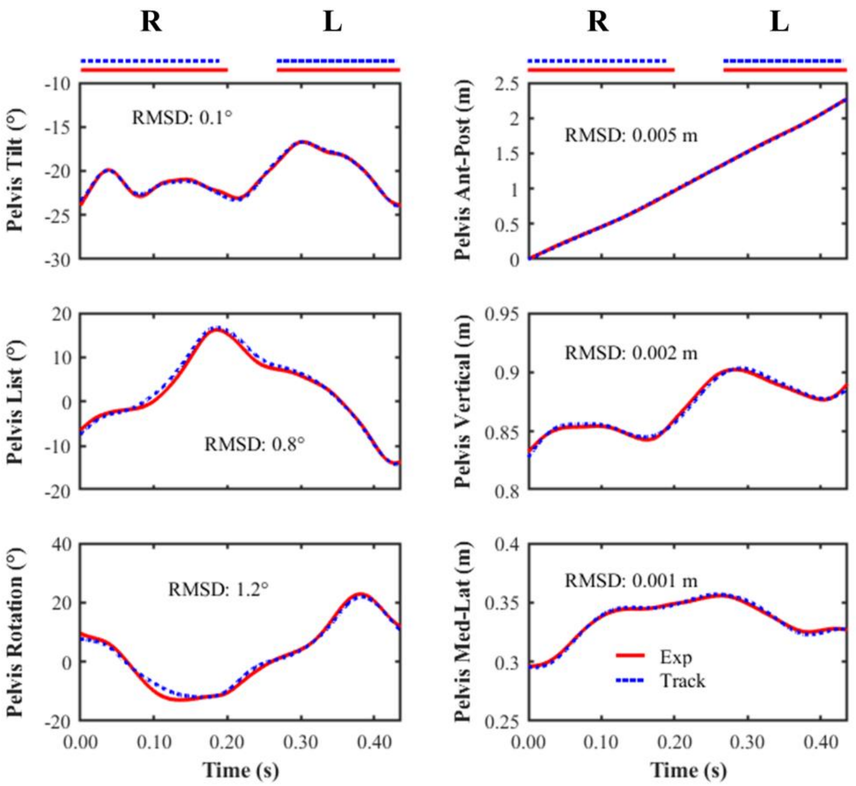

Figure S3 Global pelvis angles and translations from right foot touchdown to left foot take-off Experimental global pelvis angles and translations are denoted by solid red lines (EXP). Simulated global pelvis angles and translations are denoted by dashed blue lines (TRACK). The horizontal bars at the top of the figure indicate the periods of right (R) and left (L) foot stance.

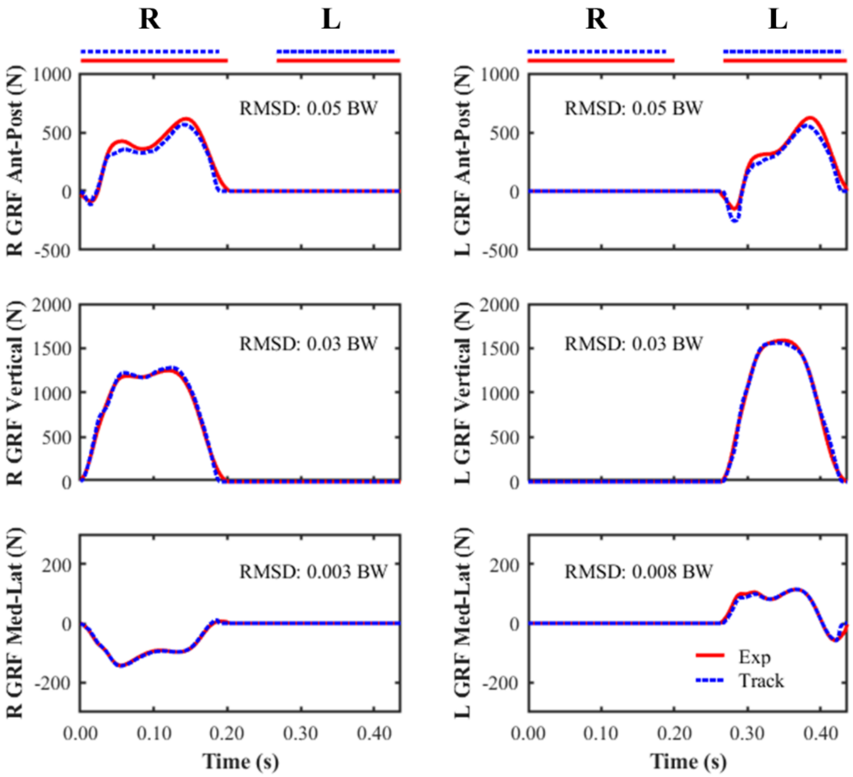

Figure S4 Right and left anterior-posterior, vertical and medial-lateral GRF from right foot touchdown to left foot take-off. Experimental GRF components are denoted by solid red lines (EXP). Simulated GRF components are denoted by dashed blue lines (TRACK). The horizontal bars at the top of the figure indicate the periods of right (R) and left (L) foot stance.

Predictive simulation results

Time histories of the ground reaction forces and pelvis kinematics for each of the predictive simulations alongside the data-tracking simulation are presented in Figures S5 and S6.

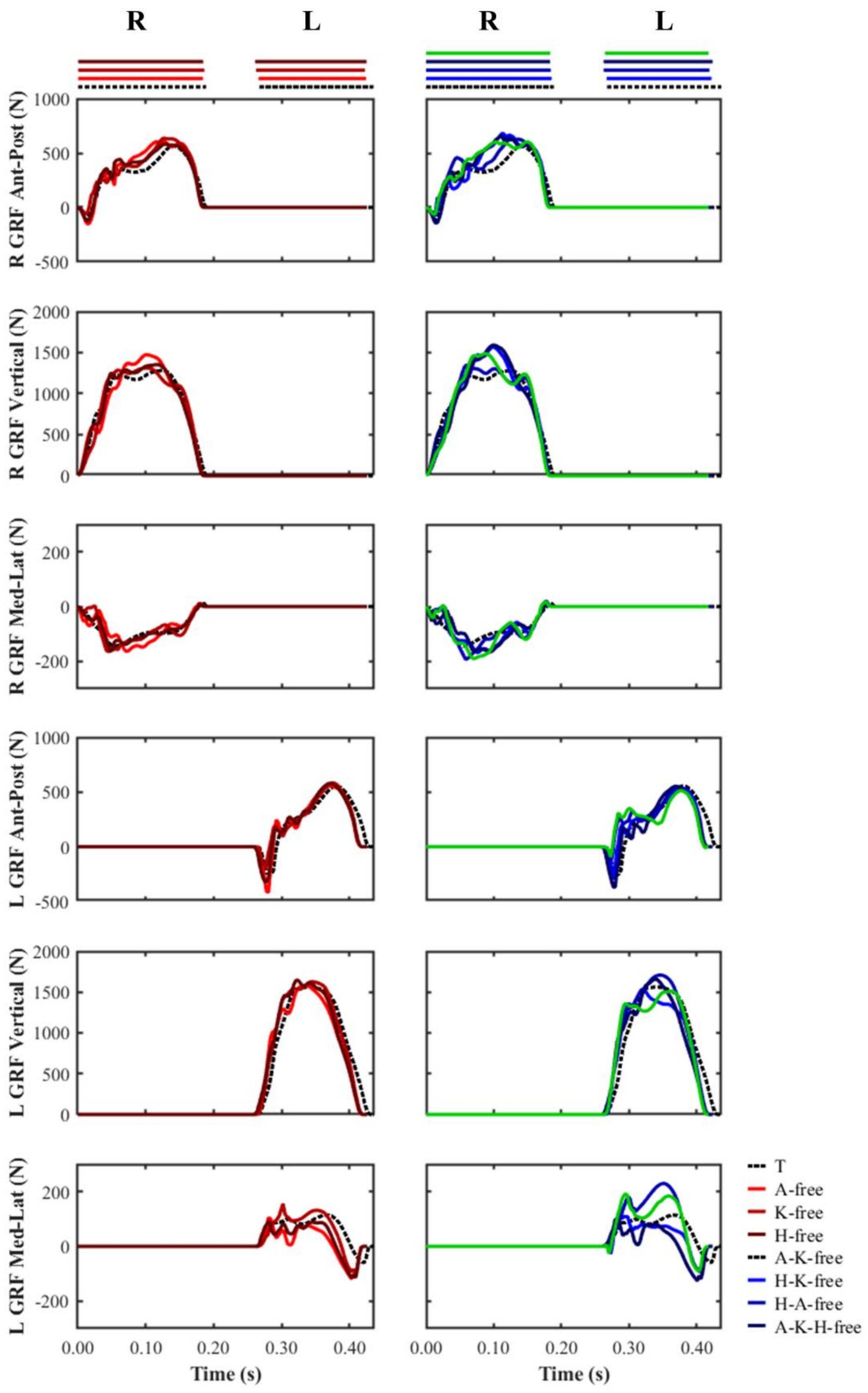

Figure S5 Right and left anterior-posterior, vertical and medial-lateral GRF from right foot touchdown to left foot take-off for the data-tracking (T) and predictive simulations (A-free, K-free, H-free, A-K-free, H-K-free, H-A-free, A-K-H-free). The horizontal bars at the top of the figure indicate the periods of right (R) and left (L) stance. The outputs from the simulations were not plotted on the same set of axes to avoid interpretability and visualisation issues.

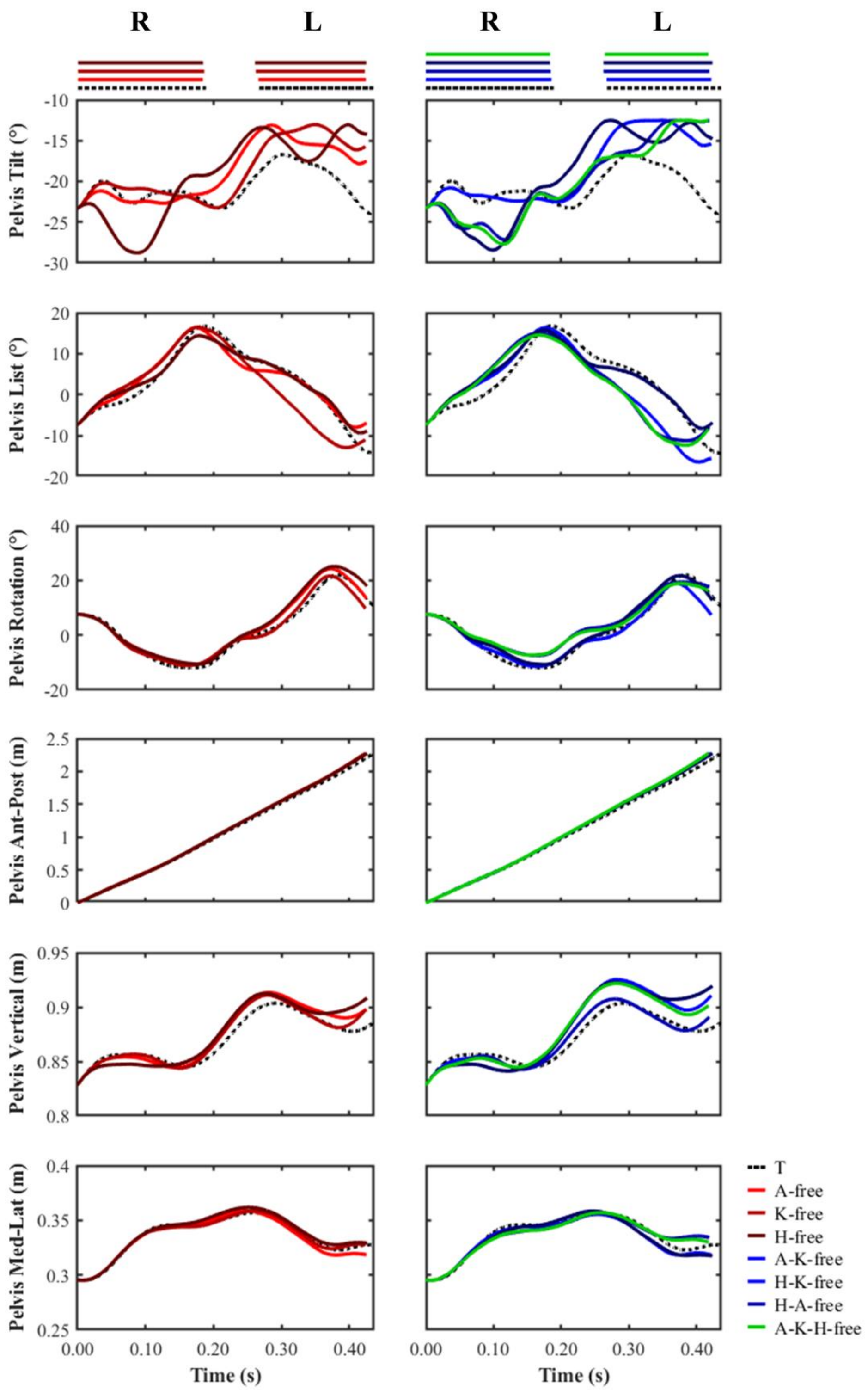

Figure S6 Global pelvis angles and translations from right foot touchdown to left foot take-off for the data-tracking (T) and predictive simulations (A-free, K-free, H-free, A-K-free, H-K-free, H-A-free, A-K-H-free). The horizontal bars at the top of the figure indicate the periods of right (R) and left (L) stance. The outputs from the simulations were not plotted on the same set of axes to avoid interpretability and visualisation issues.
